# Microstructure, Adhesion and Tribomechanical Performance of Zn–Mg–Ca- and Zn–Zr-Based Phosphate Conversion Coatings on Ti6Al4V Alloy

**DOI:** 10.3390/ma19194058

**Published:** 2026-09-23

**Authors:** Diana-Petronela Burduhos-Nergis, Nicoleta-Monica Lohan, Elena Matcovschi, Gheorghe Badarau, Costica Bejinariu, Marcelin Benchea, Fabian-Cezar Lupu, Nicanor Cimpoesu

**Affiliations:** 1 Faculty of Material Science and Engineering, Gheorghe Asachi Technical University of Iasi, 41, Prof. Dr. doc. D. Mangeron str., 700050 Iasi, Romania; diana-petronela.burduhos-nergis@academic.tuiasi.ro (D.-P.B.-N.); nicoleta-monica.lohan@academic.tuiasi.ro (N.-M.L.); elena.matcovschi@academic.tuiasi.ro (E.M.); gheorghe.badarau@academic.tuiasi.ro (G.B.); costica.bejinariu@academic.tuiasi.ro (C.B.); 2 Academy of Romanian Scientists, Ilfov 3, 050044 Bucharest, Romania; 3 Faculty of Mechanical Engineering, Gheorghe Asachi Technical University of Iasi, 61-63, Prof. Dr. doc. D. Mangeron str., 700050 Iasi, Romania; marcelin.benchea@academic.tuiasi.ro (M.B.); fabian-cezar.lupu@academic.tuiasi.ro (F.-C.L.)

**Keywords:** Ti6Al4V, biomedical implants, phosphate conversion coating, tribomechanical behavior, scratch resistance, microindentation, surface engineering

## Abstract

Ti6Al4V remains one of the most widely used metallic biomaterials for orthopedic and dental implants; however, the long-term performance of implantable devices is strongly influenced by the mechanical integrity and surface stability of the implant–tissue interface. In this study, novel phosphate conversion coatings based on Zn–Mg–Ca, Zn–Zr–Mg, and Zn–Zr–Ca systems were developed on Ti6Al4V substrates through a chemical conversion process and investigated from a tribomechanical perspective. The coatings were designed to incorporate biologically relevant elements capable of modifying crystal growth, coating compactness and interfacial characteristics. Surface morphology, coating architecture and elemental distribution were evaluated using scanning electron microscopy and energy-dispersive spectroscopy, while surface roughness was determined by profilometry. The mechanical response of the conversion-modified surfaces was assessed through instrumented microindentation and progressive-load scratch testing coupled with acoustic emission monitoring. Significant differences in coating morphology, thickness and compactness were observed as a function of phosphating chemistry. Dense and homogeneous coatings exhibited improved hardness, higher elastic recovery and enhanced resistance to plastic deformation, whereas heterogeneous structures containing large intergranular regions showed inferior mechanical performance. Scratch testing demonstrated that coating composition strongly influences damage initiation, interfacial stability and resistance to coating detachment. Among the investigated systems, Zn–Zr–Ca and selected Zn–Zr–Mg formulations provided the most favorable combination of compact microstructure, mechanical integrity and scratch resistance. The results establish clear relationships between phosphating chemistry, coating architecture and tribomechanical behavior, providing valuable insight for the design of conversion-treated Ti6Al4V surfaces intended for biomedical implant applications.

## 1. Introduction

Titanium and its alloys are among the most widely used materials in orthopedics and dentistry, thanks to their combination of biocompatibility, relatively low elastic modulus, and relatively good corrosion resistance. However, the titanium oxide layer formed on the alloy’s surface, which serves to protect the implant from interaction with biological fluids, is insufficient under real-world conditions [1]. The wear phenomenon occurring at the bone–implant interface leads to a reduction in the stability of the oxide layer, resulting in potentially toxic corrosion products that cause implant degradation and the onset of inflammatory processes in the body [2]. Consequently, to enhance the long-term performance of the implant, coating methods and surface treatments are being studied to improve both corrosion resistance and wear resistance.

To date, there have been numerous studies aimed primarily at improving the properties mentioned above. Among the most frequently studied coating methods are nitriding [3], physical and chemical vapor deposition (PVD, CVD) [4,5], plasma electrolytic oxidation [6], cold spray [7], sputtering [8], sol-gel [9], etc. Thus, it has been observed that tribological properties depend on the chemical composition of the coatings, the manufacturing process, the surface condition, and the microstructure [10]. Wozniak et al. [11] studied the influence of laser texturing on the tribological properties of the Ti6Al4V titanium alloy. Although they demonstrated an improvement due to increased microhardness and the texture’s ability to retain wear particles, performance depends on process parameters, and wear mechanisms are still present. Berni et al. [12] conducted a review in which they outlined the advantages and disadvantages of PVD coatings on titanium alloys. Although PVD may be a promising method for improving the properties of titanium alloys, they noted that it still has some major drawbacks, as the characteristics of the resulting coatings depend on process parameters. For example, the adhesion of the coating is influenced by its thickness, posing a risk of stress development and subsequent delamination. Qin et al. [13] studied the micro-arc oxidation method, a technique that generally requires improvements in terms of structure, as it often results in a highly porous, structurally unstable layer caused by the plasma discharge mechanism, as well as the presence of internal cracks that can lead to delamination. Okokpujie et al. [14] conducted a comprehensive study on various coating methods that can influence the chemical, mechanical, and tribological properties of metals. Thus, they observed that there are several limitations that may arise when considering a coating method, such as the risk of delamination or residual stresses, layer brittleness and porosity, uneven thickness, the risk of corrosion, as well as the cost and complexity of the process.

In recent years, one of the methods that has attracted attention for improving the properties of biocompatible materials is the chemical conversion process [15,16]. Compared to other methods, chemical conversion coatings have been reported to improve corrosion resistance, wear resistance and surface functionality. Depending on coating composition, some systems have also shown promising biological responses in previous studies; however, these effects remain strongly dependent on coating chemistry and testing conditions [17,18]. Regarding these types of coatings, there are relatively few studies analyzing the tribological characteristics of phosphate layers obtained through chemical conversion, most of which were conducted on steel rather than on biocompatible materials. Zang et al. [19] studied the effect of the thickness of the manganese phosphate coating deposited on steel on tribological characteristics, observing that the best performance was exhibited by a coating approximately 10 µm thick. At the same time, an improvement in the tribological characteristics of these coatings was observed following lubrication with oil [20]. Also on steel, Saffarzade et al. [21] studied the tribological behavior of magnesium phosphate-based coatings compared to that of zinc phosphate, noting that their performance depends on the degree of lubrication. Most studies focus on the coefficient of friction (COF), which should be lower than that of the substrate, but it depends on the roughness of the coating. However, when phosphate layers deposited by chemical conversion are intended for use in biomedical applications, the comparison between COF values is no longer the primary concern; rather, it is important that the behavior becomes relatively stable and that there is no delamination or massive wear, thereby limiting significant degradation of the substrate. Regarding chemically converted phosphate coatings on biomaterials, studies focus on improving corrosion resistance and promoting osseointegration, without emphasizing their tribological behavior. Despite the growing interest in phosphate conversion coatings for titanium-based implants, current research has been predominantly focused on improving corrosion resistance, antibacterial performance, and osteogenic activity through the incorporation of bioactive ions such as Ca, Mg, Zn and Sr. Recent studies have demonstrated that phosphate-based coatings can promote biological responses and enhance surface functionality; however, the relationship between coating chemistry, microstructural development, and tribological performance remains insufficiently understood, particularly for Ti6Al4V substrates. Most published investigations emphasize biological or electrochemical behavior, while systematic analyses of local mechanical response, adhesion mechanisms, scratch-induced damage evolution, and coating/substrate interfacial stability are scarce. In addition, no study has been reported, to the best of the authors’ knowledge, on tricationic phosphate conversion systems based on Zn–Mg–Ca, Zn–Zr–Mg, and Zn–Zr–Ca deposited on Ti6Al4V through chemical conversion processing. Therefore, the novelty of the present work lies not only in the development of these original phosphating formulations, but also in the establishment of a direct structure–property relationship between ionic composition, crystal morphology, and tribological performance. To achieve this objective, a multiscale characterization approach combining cross-sectional SEM/EDS analysis, roughness measurements, instrumented microindentation, progressive-load scratch testing with acoustic-emission monitoring, and post-test SEM examination was employed. This methodology provides new insight into the micromechanical and tribological behavior of chemically converted phosphate coatings, and identifies the compositional parameters governing coating integrity and resistance to deformation and wear under localized contact loading.

This study focuses on the tribological behavior of nine types of phosphate coatings, with different chemical compositions and morphologies, deposited by chemical conversion onto the surface of a Ti6Al4V alloy. Consequently, the influence of coating morphology on their tribological characteristics can be analyzed. The phosphate coatings analyzed are trivalent and have not been previously reported in the literature; the chemical composition of the phosphating solutions used is original, allowing the authors to study the influence of ions on the coating properties, with three systems investigated: Zn–Mg–Ca, Zn–Zr–Mg, and Zn–Zr–Ca. The paper presents results for the representative sets (I.1, II.3, and III.2); however, all results obtained from the sample sets are discussed. The coatings were designed for potential biomedical applications, but the present work evaluates only structure, micromechanical behavior, scratch resistance and tribological performance.

## 2. Materials and Methods

### 2.1. Materials

The Ti6Al4V alloy used as the coating substrate was purchased from AEMMetal Company (Changsha, China). According to the supplier’s compositional data, the alloy contained, in wt.%, 6.14 Al, 4.22 V, 0.11 Fe, 0.18 O, 0.028 C, and Ti as the balance. Because certification according to a specific biomedical material standard was not available, the material is referred to as Ti6Al4V rather than medical-grade Ti6Al4V.

### 2.2. Chemical Conversion Process (Phosphating)

The Ti6Al4V specimens (2.5 × 2.5 × 20 mm) were mechanically prepared by grinding to 1200 grit, after which they were cleaned and chemically activated, as shown in Figure 1. Subsequently, they were immersed in the phosphating solution for 60 min at 90 °C. Nine types of coatings were obtained and analyzed using phosphating solutions with different chemical compositions. These were divided into three sets, as shown in Table 1, based on different percentages of the components. The chemical composition of the phosphating solution was calculated for 1 L. The pH of the solution varies between 3.00 and 3.50 and was adjusted using 10M NaOH solution. Before the phosphating experiments, each freshly prepared solution was conditioned by immersing a 5.00 g piece of metallic Fe for 24 h. The Fe piece was subsequently removed, and the Ti6Al4V specimens were then introduced into the conditioned bath for phosphating.

Prior to phosphating, the Ti6Al4V substrates underwent a sequential surface preparation procedure consisting of ultrasonic degreasing for 10 min to remove contaminants, followed by chemical pickling in a 2% HF solution for 15 s to eliminate the native oxide layer and increase surface reactivity. The samples were then activated in a solution containing 3 g/L colloidal titanium for 30 s, promoting the formation of nucleation sites for phosphate crystal growth. After activation, the specimens were immersed in the phosphating bath at 90 °C for 60 min, allowing the development of a conversion coating composed primarily of phosphate species and metal cations present in the solution. Upon completion of the treatment, the coated samples were thoroughly rinsed with deionized water to remove residual reaction products, and subsequently dried before characterization. The resulting phosphate layers were evaluated in terms of morphology, elemental composition, surface roughness, scratch resistance, and microhardness using SEM/EDX analysis, profilometry, scratch testing, and microindentation techniques. The formation of the phosphate layers follows the classical mechanism of chemical conversion coatings, in which local dissolution of the activated titanium surface is coupled with the precipitation of insoluble phosphate compounds. Following HF activation, the removal of the native TiO_2_ film increases the electrochemical activity of the substrate and promotes localized anodic dissolution. Simultaneously, cathodic reactions occurring on the titanium surface increase the local pH at the metal/solution interface, creating favorable conditions for phosphate precipitation.

The presence of Zn^2+^, Mg^2+^, Ca^2+^, and Zr-containing species modifies both the supersaturation level and the nucleation kinetics. Zn^2+^ ions predominantly promote the formation of hopeite-type zinc phosphate crystals, whereas Mg^2+^ and Ca^2+^ partially substitute within the phosphate structure and alter crystal growth rates. Zirconium species act as heterogeneous nucleation promoters and growth inhibitors, increasing nucleation density while limiting excessive crystal coarsening. Consequently, the final coating architecture is governed by the competition between nucleation and crystal growth processes, leading to either coatings that are too thick composed of large lamellar crystals, or to thinner and more compact layers characterized by finer crystalline assemblies.

### 2.3. Characterization Methods

The morphology of the conversion coatings before and after scratch tests was studied using a Vega Tescan LMH II (SE detector, 30 kV, TESCAN, Brno, Czech Republic) scanning electron microscope (SEM). The chemical composition and the coatings’ elemental distribution were determined using an energy-dispersive detector (EDX Bruker, Berlin, Germany). Qualitative pseudo-three-dimensional representations of selected scratch tracks were generated from secondary-electron SEM images using the 3-D View function of the VegaTC software Esprit 2.2 associated with the Vega Tescan LMH II microscope. The software converts variations in SE-image grayscale intensity into an apparent relief representation. Corresponding intensity profiles were extracted along selected paths across the scratch tracks. Because SE intensity also depends on detector geometry, specimen composition, edge effects, and charging, these reconstructions and profiles were used only for the qualitative visualization of surface heterogeneity and scratch-track morphology. They do not represent calibrated surface heights or quantitative groove depth measurements.

The morphological characteristics and thickness of the phosphate coatings were investigated using a Quattro S Scanning Electron Microscope (SEM, Thermo Fisher Scientific, Waltham, MA, USA). To ensure a representative statistical distribution, cross-sectional micrographs were captured at a magnification of 500× using a Large Field Detector (LVD) in Secondary Electron (SE) mode (Thermo Fisher Scientific, Waltham, MA, USA), with an accelerating voltage of 20.00 kV and a working distance (WD) maintained between 9.7 mm and 10.9 mm. The thickness of the deposited layers was determined by performing multiple point-to-point measurements (four per micrograph) across the interface between the substrate and the coating, using the SEM’s integrated measurement software.

The thickness and chemical depth profiles of the newly developed phosphate coatings (Sets I, II, and III) were evaluated through cross-sectional scanning electron microscopy (SEM) and energy-dispersive X-ray spectroscopy (EDS) point analysis. Cross-sectional EDS point analysis was performed at six selected positions for each specimen to examine specific regions of the heterogeneous coating–substrate cross-section. Points 1–3 were located within the phosphate coating, whereas points 4–6 were positioned near the interface and progressively toward the bulk Ti6Al4V substrate. This approach enabled localized, semi-quantitative compositional comparisons among visibly distinguishable regions while limiting signal averaging across pores, intercrystalline spaces, and irregular interfaces. For sample I.1, the six measurement positions covered a total cross-sectional distance of 14.22 μm, as indicated in Figure S1; however, the spacing between consecutive points was not uniform, and the exact point-to-interface distances were not recorded. Consequently, the measurements provide local compositional comparisons rather than a spatially continuous concentration profile, and cannot independently establish elemental diffusion, quantify penetration depth, or determine the width of a chemically modified interfacial region. Signals detected near the interface may also be influenced by the EDS interaction volume, interface roughness, coating porosity, specimen preparation, and exact point placement. Confirmation of an elemental concentration gradient would require distance-calibrated line scans or elemental maps, preferably supported by higher-resolution techniques such as EPMA/WDS, TEM–EDS/EELS, SIMS, or another suitable depth-profiling method. The demonstration of a corresponding mechanical gradient would additionally require spatially resolved low-load nanoindentation across the coating–substrate interface.

Surface roughness was evaluated using a Taylor–Hobson Talysurf I50 profilometer (Taylor Hobson Ltd., Leicester, UK) over a probe travel length of 10 mm. Three measurements were performed at different surface positions for each sample, and the results are reported as mean ± standard deviation. A chisel edge stylus arm (112/2013) and an inductive gauge sensor were employed for all profilometric analyses.

The scratch resistance and adhesion of the phosphate layer deposited on the sample surfaces were assessed by microscratch testing using a CETR-UMT-2 tribometer (Campbell, Logan, UT, USA). This technique involves a sharp stylus producing a controlled and continuous scratch on the material surface. Acoustic emission (AE) signals were simultaneously recorded to provide information on the load–displacement behavior during scratching, enabling the identification of damage initiation and failure mechanisms. Two microscratch test modes were employed: constant-load and progressive-load (linearly increasing force) tests. All experiments were performed under a maximum normal load of 10 N. During testing, the sample stage was displaced over a distance of 10 mm in 60 s, corresponding to a scratching speed of 0.167 mm/s. A cutting blade stylus (Nvidia, Santa Clara, CA, USA) with a tip radius of 0.4 mm was used for the microscratch experiments.

Scratch-induced damage was classified into cohesive and adhesive modes. Cohesive damage comprised microcracking, crushing, and fragmentation within the phosphate crystal network without clear separation from the substrate. Adhesive damage was assigned only when post-test SEM observations showed local interfacial detachment, lateral delamination, or exposure of the Ti6Al4V substrate. Crystal fragmentation, ploughing, and debris formation alone were not considered evidence of adhesive failure. Because no synchronized in situ microscopic monitoring was employed, Lc1, Lc2, and Lc3 represent qualitative estimates obtained by correlating acoustic emission and friction signal variations with post-test SEM/EDS observations.

The effective hardness of the coated surface system was evaluated by microindentation testing using a Rockwell diamond indenter with a tip radius of 200 μm and an apex angle of 120°. For each sample, three microindentation tests were performed under a normal load of 10 N. Three samples from each of three different batches were analyzed. All experimental data were processed and interpreted using Test Viewer software, version 2.16. To assess possible substrate influence, the maximum indentation displacement, hmax, was compared with the measured coating thickness, t. A commonly employed guideline for determining intrinsic coating properties requires the penetration depth to remain below approximately 10% of the coating thickness hmax/t ≤ 0.1. In the present study, hmax ranged from 4.33 to 13.22 μm, while coating thickness ranged from approximately 3.43 to 20.59 μm. Thus, this criterion was not satisfied, indicating a substantial contribution from the Ti6Al4V substrate. Consequently, the calculated hardness and effective elastic modulus of the coated surface system are interpreted as effective values characterizing the combined coating–interface–substrate response rather than the intrinsic properties of the phosphate layer.

Samples I.1, II.3, and III.2 were selected for detailed graphical presentation because they represent the three investigated chemical systems and illustrate characteristic differences in coating morphology and tribomechanical response. Sample I.1 exhibited comparatively compact coverage and the highest scratch-track integrity within Set I, sample II.3 combined the lowest roughness in Set II with an intermediate deformation–fragmentation response, and sample III.2 showed the most compact and homogeneous morphology together with the most favorable overall mechanical and scratch resistance behavior. The selection was made only to provide clear representative examples; the results obtained for all nine coatings were included in the comparative analysis and are reported in the main text and Supplementary Materials.

Although the coatings contain biologically relevant elements such as Zn, Mg, Ca, and Zr, their bioactivity cannot be inferred solely from their elemental composition and was not demonstrated in the present study. No electrochemical, ion release, or biological investigations were performed. Therefore, the present results establish only the structural and tribomechanical characteristics of these coatings and their potential for subsequent biomedical evaluation. The confirmation of biomedical suitability will require corrosion and ion release studies in physiologically relevant media, followed by appropriate in vitro and in vivo biological assessments.

## 3. Results and Discussions

The comparative analysis of the phosphate coatings reveals a clear correlation between the chemical formulation of the precursors and the resulting layer structure. The significantly higher thickness and associated standard deviation observed in sample I.2 (20.59 ± 7.12 μm) suggest a rapid, possibly dendritic growth mechanism, likely leading to a more porous structure, which may influence load transfer, surface roughness and tribological response. In contrast, the transition to the Series II samples marks a refinement of the deposition process, characterized by a substantial reduction in thickness and a more compact morphology. Specifically, sample II.2 (3.43 ± 0.53 μm) represents the most refined coating, where the low standard deviation indicates a high nucleation density and a suppressed vertical growth rate. This structural densification is often associated with the incorporation of elements like magnesium or zirconium, which can alter the crystallization kinetics of the zinc phosphate phase. Furthermore, the intermediate values obtained for Series III suggest a balanced growth regime, providing a compromise between the robust coverage seen in Series I and the structural integrity of Series II. These variations in thickness and uniformity are expected to influence the mechanical and tribological performance of the conversion-modified surface, and may also affect corrosion behavior; however, corrosion performance was not evaluated in the present study.

Cross-sectional EDS point analysis revealed local compositional differences among the phosphate coating, near-interface region, and Ti6Al4V substrate. Coating-associated elements were detected at some positions near the interface; however, these localized measurements do not establish a continuous concentration profile or confirm solid-state diffusion into the substrate. For sample III.2, point 6 was dominated by Ti and Al, with concentrations of 76.0 and 20.6 at.%, respectively, whereas only trace amounts of P (0.2 at.%) and Zn (0.6 at.%) were detected. These trace signals cannot be interpreted as evidence of elemental penetration or a diffusion depth of approximately 20 μm because they may be influenced by the EDS interaction volume, interface roughness, coating porosity, specimen preparation, and exact point placement. Consequently, the results are interpreted only as localized, semi-quantitative compositional measurements. The confirmation of an elemental gradient would require distance-calibrated line scans, elemental maps, or an appropriate depth-profiling technique. Accordingly, the results are interpreted only as evidence of local compositional variation across the interfacial region (Figures S1–S9 and Tables S1–S9).

### 3.1. The Micromorphology and Chemical Composition of the Phosphate Conversion Coatings

Differences in the morphology of the phosphate layers influence both their surface roughness and their tribological behavior. Figure 2 shows the morphology of the phosphate layers obtained with phosphate-based solutions (see Table 1). As can be seen from the images, the morphology of the layer is heterogeneous. The crystals formed on the entire surface of Ti6Al4 are lamellar, well-developed and randomly oriented. Additionally, relatively large intercrystalline zones are observed, exhibiting a moderately compact structure. Analyzing the results compared to I.1, layer I.2 exhibits a morphology consisting of interconnected polyhedral crystals, as well as zones with crystalline aggregates, thus forming a highly compact structure. The SEM analysis of layer I.3 indicates a more uniform coverage of the substrate, with a finer polycrystalline structure, featuring fewer and smaller intercrystalline spaces compared to the other two samples. The surface chemical composition of the representative phosphate coatings was determined by EDS over three different areas of 1 mm^2^ for each sample. The results presented in Table 2 are expressed as mean values with the corresponding standard deviations calculated from these three area measurements. Based on the phosphating bath composition, EDX data and previous reports on zinc–phosphate conversion coatings, the deposited layers are likely to contain zinc–phosphate species, potentially including hopeite-type phases reported in similar systems [18], (Zn_3_(PO_4_)_2_·4H_2_O), accompanied by minor Mg- and Ca-containing phosphate phases and, where applicable, zirconium phosphate/zirconia species. At the coating–substrate interface, titanium oxide and titanium phosphate species are likely formed, producing a chemically graded Ti–O–P transition zone.

The coating from sample I.1 exhibits the highest concentrations of Zn and P, indicating the formation of a layer rich in zinc phosphate, with very low contributions from Mg and Ca. At the same time, the low presence of substrate-specific elements (Ti, Al, V) indicates the degree of substrate coverage, with the layer exhibiting high compactness and very few intergranular zones, consistent with the crystalline morphology observed under SEM. As with the other two samples from set I (I.2 and I.3), the concentrations of Zn, P, and O suggest the formation of a phosphate layer; however, in this case, the morphology is influenced by the presence of Ca and Mg in greater quantities than in I.1, a feature also observed in the SEM. Furthermore, the amount of Ti in the other two samples (I.2 and I.3) is significantly higher than in I.1, indicating more numerous and larger intergranular zones, as well as a thinner layer, particularly in the case of layer I.3. Thus, it can be concluded that layer I.1 exhibits a structure dominated by zinc phosphate crystals, their overlapping resulting in better coverage of the substrate, whereas the influence of Mg, and of Ca for the other two samples, alters the shape and size of the crystals, leading to a structure with higher porosity.

The morphology of the Zn–Zr–Mg-based samples differs from that of the Zn–Mg–Ca-coated samples (Figure 2). Thus, layer II.1 exhibits a structure composed of lamellar crystals or thick, overlapping plates oriented in different directions (Figure S4). At the same time, at high magnifications (500×), agglomerations of fine particles can be seen, which are preponderately localized on the crystal surface. The layer presents a compact structure, but some heterogeneous zones, which can influence the roughness and tribological behavior of the sample. Compared to sample II.1, the layer specific to II.2 exhibits a morphology consisting of a higher density of short lamellar or prismatic crystals, arranged randomly—Figures S4 and S5. Furthermore, the crystal sizes are smaller, their growth being inhibited by the greater number of crystallization centers. In the case of sample II.3, although the crystals have the same shape as in sample II.2 (Figure S5), they are better defined, arranged radially, and interconnected. Their increase in size indicates a lower number of crystallization centers, thus accounting for the presence of more visible intergranular spaces. Regarding the EDX analysis of the coating (Table 2), as expected, the highest Zn content is found in sample II.1, while the Mg and Zr contents in the other two samples (II.2 and II.3) vary with their concentrations in the phosphating solution—Tables S4 and S5. Sample II.1 has the fewest substrate-specific elements due to the layer’s thickness. Sample II.2, although it has more crystals, has a thinner layer and more micro-intergranular zones, due to their small size, which explains the slight increase in Ti content—Figure S5. The intergranular spaces visible in sample II.3 account for the highest amount of substrate elements.

As observed so far, the morphology of the phosphate layer is influenced by the metal ions added to the phosphating solution. Thus, the shapes and distributions of the crystals differ in set III (Figure 2). Sample III.1 exhibits a heterogeneous structure, with elongated, lamellar, or acicular crystals, which are well-defined and oriented in different directions—Figure S7. The compactness of the layer is moderate, with many visible intercrystalline areas. At 500×, small, fine crystals can be observed in the intergranular zones, indicating continuous nucleation and uneven growth. In the case of sample III.2, the layer’s morphology is completely different, being more uniform and showing better compactness. The layer consists of shorter, lamellar or acicular crystals, exhibiting smaller intergranular zones. The morphology of sample III.3 consists of irregular clusters of crystals and much larger intercrystalline zones than in the other samples—Figure S9. The crystals are thicker and, in certain areas, appear fused together, indicating uncontrolled growth. The DX analysis (Table 2) of sample III.1 confirms the formation of a phosphate layer, as all elements characteristic of it P, O, Zn, Zr and Ca are present—Table S7. Although the crystals are well developed, the Ti content is quite high (20 wt.%), indicating moderate compactness. The EDX results for sample III.2 confirm the layer’s greater compactness compared to the other two (III.1 and III.3), with the lowest Ti percentage (10.8 wt.%). The layer with the largest intergranular zones and the greatest substrate exposure is sample III.3, where EDX analysis revealed decreases in P, O, and Zn and an increase in Ti content (37.7 wt.%). Of all nine samples analyzed, this is the one with the lowest compactness—Tables S7 and S9.

The observed morphological differences can be interpreted in terms of nucleation density and crystal growth competition during phosphating. Coatings rich in Zn and P, such as specimen I.1, favor rapid crystal growth after nucleation, resulting in large overlapping phosphate crystals and increased coating thickness. The addition of Mg and Ca modifies the precipitation kinetics by increasing the number of nucleation sites and reducing preferential crystal growth, producing finer crystal assemblies but also increasing the number of intergranular boundaries. In the Zn–Zr-containing systems, zirconium species appear to suppress excessive crystal growth while promoting a larger number of nucleation events, which explains the refined morphology observed for specimen II.2. As a result, coating architecture evolves from a crystal growth-dominated structure (large crystals, higher roughness and larger intergranular voids) toward a nucleation-dominated structure (smaller crystals, higher compactness and reduced surface heterogeneity). The observed architectural evolution was accompanied by localized compositional differences across the coating–substrate cross-section. However, the discrete EDS measurements cannot establish a chemically graded interface or its contribution to stress redistribution.

### 3.2. Microindentation Tests

The average values of the parameters obtained from the microindentation tests, along with the load-displacement curves, are presented in Table 3 and the Figure S10, respectively. By analyzing the values of the effective hardness of the coated surface system (H), the indentation Young’s modulus (E) and the ratios between them, it can be observed that the highest values correspond to specimen I.3, while the value of h_max_ for the same specimen is the lowest. This indicates superior resistance to deformation compared to sample I.2, where the values indicate that the layer deforms easily, exhibiting lower mechanical strength. At the same time, indentation E suggests that layer I.3 is stiffer, being more resistant to elastic deformation. The contact depth values are consistent with the H values, being lower when H and h_max_ are higher. For the samples in set I, the values are influenced by the layer’s morphology, with contact depth being smaller for more compact or finer layers, as these offer greater resistance to indenter penetration. The H/E ratio was calculated to provide information about the layer’s ability to recover after the force is removed and its resistance to elastic deformation, while the plastic deformation index (H^3^/E^2^) helps evaluate the layer’s resistance to plastic deformation under the action of a force. Once again, sample I.3 exhibits better performance, as it withstands greater elastic deformations and recovers after deformation. In addition, the higher H^3^/E^2^ value for I.3 indicates that the material exhibits greater resistance to plastic deformation than the other two specimens. It should be emphasized that the microindentation results do not represent the comparative indentation response of the phosphate crystals alone. Because the indentation depth exceeded 10% of the coating thickness, the microindentation results represent the effective mechanical response of the combined coating–substrate system rather than the intrinsic response of the phosphate crystals. The measured values were therefore influenced by coating thickness, compactness, crystal morphology, structural continuity, and the underlying Ti6Al4V substrate. This approach is appropriate for conversion coatings, where adhesion and load transfer are governed by the continuity between the precipitated phosphate layer and the chemically affected substrate surface.

The hardness and effective elastic modulus of the coated surface system values presented in Table 3, Table 4 and Table 5 must be interpreted considering the substantial substrate contribution. For every coating, the maximum penetration depth exceeded 10% of the measured layer thickness. Accordingly, differences among specimens reflect the combined effects of coating thickness, compactness, structural continuity, interfacial response, and support from the Ti6Al4V substrate. This limitation is particularly important for thin or discontinuous coatings, such as III.3, for which values approaching those of uncoated Ti6Al4V primarily indicate substrate-dominated behavior, and should not be interpreted as superior intrinsic coating properties [22].

The results of the profilometric analysis for sample I.2 show that it has the most uneven surface, which is fully consistent with the low values of the microindentation parameters, indicating both low hardness and low resistance to elastic and plastic deformation.

When comparing the values of the layers in Set I with those of the uncoated Ti6Al4V sample [23], it is observed that the latter exhibits the best mechanical properties. The hardness and elastic modulus of the uncoated titanium alloy are significantly higher than those of the Zn–Mg–Ca layers, indicating the material’s high stiffness. At the same time, it exhibits the smallest contact depth and the highest H/E ratio, suggesting its ability to withstand contact stresses without local damage. All of this indicates that sample I.2 is the most deformable layer, I.1 exhibits intermediate behavior, and I.3 is the most mechanically resistant of all the deposited layers. The results for the Zn–Mg–Ca samples are fully consistent with the SEM observations (Figures S2 and S3)—sample I.2 exhibits the most uneven morphology, with multiple intergranular zones, and displays the poorest mechanical properties (Figure S2). These findings suggest that the structure and chemical composition of the phosphate layer influence its mechanical behavior.

The average values for the Zn–Zr–Mg-based coatings obtained from the micro-indentation tests are given in Table 4, showing that the effective hardness of the coated surface system of specimen II.1 is lower than that of the substrate, indicating that the layer may be easily affected by local mechanical stresses [23]. This observation is further supported by the values of h_max_ and E, as the layer undergoes greater elastic deformation than the substrate. Furthermore, the contact depth is close to h_max_, indicating that a large portion of the total deformation corresponds to effective penetration into the material, which has limited resistance to indenter penetration.

The H/E ratio indicates that the layer readily transitions to a plastic deformation regime under mechanical stress. At the same time, H^3^/E^2^ indicates that the layer does not yield immediately but does not exhibit resistance to plastic deformation. In sample II.2, the effective hardness of the coated surface system value is significantly higher, indicating a layer that is more resistant to penetration. At the same time, the maximum displacement and contact depth confirm that the layer is stiffer and more mechanically resistant than layer II.1. The compact, more uniform structure of layer II.2 is also confirmed by the elastic stiffness, which is higher in this case. The ratios of H to E indicate that sample II.2 can withstand greater elastic deformation before plastic deformation occurs, indicating better resistance to plastic deformation and wear. These results are consistent with both the profilometric analysis values and the layer’s morphology. Layer II.3 exhibits behavior similar to that of sample II.1, though slightly lower. The values for contact stiffness, maximum displacement, and contact depth are higher than those of the other two samples. Thus, it appears that the layer offers little resistance to penetration, although it is more uniform. Of the three samples, layer II.3 is the least resistant to plastic deformation. The results of the microindentation tests for the specimens in Set III are presented in Table 5.

The average effective elastic modulus of the coated surface system for sample III.1 indicates moderate, relatively stable elastic stiffness, a finding supported by the contact stiffness value. The effective hardness of the coated surface system of sample III.1 is relatively low, characteristic of a phosphate layer with moderate compactness, as further supported by the relatively high contact depth. The H/E ratio suggests that the layer transitions readily from elastic to plastic deformation. At the same time, the H^3^/E^2^ value is consistent with the low hardness, suggesting limited resistance to plastic deformation. Due to the compactness of the layer and its morphology, sample III.2 exhibits higher hardness than sample III.1, as evidenced by the significantly lower contact depth. At the same time, the sample exhibits greater elastic stiffness and better resistance to elastic stresses. Furthermore, it also exhibits much better resistance to plastic deformation. The high hardness and effective elastic modulus values obtained for the coated surface system of sample III.3 are very close to those of Ti6Al4V; this result reflects the behavior of the substrate and the presence of a surface layer. The result is consistent with the morphology of layer III.3, which is discontinuous and contains large crystalline zones, allowing the indenter to interact directly or partially with the substrate.

Figure 3 shows differences between the effective hardness of the coated surface system and Young’s modulus values of the uncoated Ti6Al4V specimen and the phosphate specimens. Thus, it can be observed that the effective hardness of the coated surface system of the coated specimens is much lower than that of Ti6Al4V, except for specimen III.3, but this does not reflect the actual effective hardness of the coated surface system of the coating. Among the other specimens, specimen III.2 has the highest hardness value. At the same time, the Young’s modulus value follows the effective hardness of the coated surface system trend, being lower for the coated samples, except III.3. Even though these values are lower than those of Ti6Al4V, when using these coatings to improve the implant’s characteristics, the obtained values can reduce the difference between the stiffness of titanium and that of bone. The microindentation results reveal that the phosphate conversion coatings deposited on Ti6Al4V exhibited effective hardness values between 0.61 and 2.19 GPa and an elastic moduli ranging from 73.15 to 156.95 GPa, depending on coating composition and morphology. The most favorable behavior among the chemically converted coatings was observed for sample III.2 (H = 1.31 ± 0.35 GPa; E = 106.62 ± 14.07 GPa), which combined high compactness, low contact depth and improved resistance to elastic and plastic deformation, while sample II.2 also demonstrated a balanced mechanical response associated with its refined and uniform microstructure. In contrast, sample I.2 displayed the lowest hardness (0.61 ± 0.05 GPa) and modulus (73.15 ± 3.89 GPa), reflecting its highly porous and heterogeneous morphology. Furthermore, the H/E and H^3^/E^2^ ratios confirmed that the denser coatings exhibited superior elastic recovery and greater resistance to plastic deformation.

The indentation response can be rationalized considering the architecture of the conversion coatings rather than solely their chemical composition. During indentation, the applied load is initially supported by the phosphate crystal network and subsequently transferred through crystal boundaries toward the substrate. In coatings containing large lamellar crystals and extensive intergranular regions (e.g., I.2 and III.1), stress concentrations develop at crystal boundaries, facilitating localized crystal rotation, boundary sliding and microcracking, which results in greater penetration depths and lower hardness values. Conversely, in compact coatings characterized by finer and more uniformly distributed crystals (II.2 and III.2), the higher density of crystal–crystal contacts promote a more homogeneous stress distribution beneath the indenter. The improved indentation response of samples II.2 and III.2 may be attributed to their greater compactness, finer crystal morphology, and more uniform distribution of load within the coating–substrate system. The EDS point measurements do not demonstrate a diffusion zone or mechanically graded interface. This architecture explains the higher H/E and H^3^/E^2^ ratios measured for II.2 and III.2, indicating improved resistance to elastic strain accumulation and a delayed onset of plastic deformation. Therefore, the indentation behavior is controlled primarily by coating compactness, crystal connectivity and interfacial stress transfer efficiency, rather than by coating thickness alone.

Compared with the literature, the effective hardness of the coated surface system values obtained in the present study are lower than those reported for dense calcium phosphate coatings deposited by advanced physical vapor deposition techniques. For example, Bai et al. reported microhardness values exceeding 5.27 GPa and Young’s modulus values above 91.7 GPa for ion-beam-assisted calcium phosphate coatings deposited on titanium, while Quirama et al. observed similarly enhanced mechanical performance for crystalline HA-based coatings deposited by RF magnetron sputtering with TiN/TiO_2_ interlayers [24]. These differences are expected because the coatings developed in the present work were obtained by chemical conversion at 90 °C, producing relatively thick crystalline phosphate layers with significant intergranular porosity and heterogeneous crystal growth, whereas sputtered or ion-beam-assisted coatings are typically thinner, denser, more highly compact and characterized by strong atomic-scale bonding to the substrate. Additionally, Saber-Samandari et al. showed that increasing substrate temperature during calcium phosphate deposition improved the effective hardness and effective elastic modulus of the coated surface system by promoting densification and reducing structural defects, confirming the critical effect of processing conditions on micromechanical properties [25,26]. The observed variations can also be explained by differences in coating architecture, crystallinity, thickness and substrate influence [27,28]. In the present work, samples with large intergranular regions and lower compactness (e.g., I.2 and III.1) exhibited higher contact depths and lower hardness, whereas the more compact coatings (II.2 and III.2) showed improved stiffness and deformation resistance. The high hardness and effective elastic modulus of the coated surface system values obtained for sample III.3 should not be interpreted as superior intrinsic coating properties. SEM and EDS results indicate a discontinuous coating architecture with considerable substrate exposure. Under the applied indentation load, the indenter response is dominated by the mechanically stronger Ti6Al4V substrate rather than by a continuous phosphate layer. Thus, III.3 represents a coating/substrate-dominated response, while the more compact coatings, particularly II.2 and III.2, provide a more representative response of the conversion-modified surface system.

The maximum load values measured during the microindentation tests remained practically constant for all investigated specimens, varying only within the experimental uncertainty range (8.99–9.02 N). This behavior is expected because the tests were performed under a controlled loading protocol with a predefined target load of approximately 9 N. Consequently, the negligible differences observed among Sets I, II, and III do not reflect intrinsic variations in coating properties, but rather confirm the excellent repeatability and stability of the testing procedure. Therefore, the differences observed in displacement, the effective hardness of the coated surface system, the effective elastic modulus of the coated surface system, and deformation-related indices can be directly attributed to the mechanical responses of the phosphate coatings and their microstructural characteristics, rather than to variations in the applied load. The reduced modulus values closely follow the evolution of Young’s modulus for all investigated coatings, reflecting the strong relationship between these two parameters. Since the reduced modulus incorporates the elastic contribution of both the indenter and the tested material, higher values indicate a greater resistance to elastic deformation under contact loading. In Set I, the reduced modulus increases from 77.14 GPa for specimen I.2 to 90.35 GPa for specimen I.3, confirming the superior stiffness of the latter coating. A similar trend is observed in Set II, where specimen II.2 exhibits the highest reduced modulus (92.78 GPa), consistent with its compact morphology and enhanced mechanical integrity. The most pronounced increase is observed in Set III, reaching 153.68 GPa for specimen III.3. However, this exceptionally high value is likely influenced by substrate participation during indentation due to the discontinuous morphology of the coating. Overall, the reduced modulus results corroborate the Young’s modulus measurements, and demonstrate that coating compactness, crystal distribution, and substrate exposure play a decisive role in determining the elastic stiffness of the phosphate conversion layers.

The contact stiffness values exhibit relatively limited variation compared with the effective hardness of the coated surface system and effective elastic modulus of the coated surface system, indicating that all coatings maintained a comparable resistance to elastic contact loading. Within Set I, specimen I.2 showed the highest contact stiffness (6.34 N/µm), followed by I.1 (6.14 N/µm) and I.3 (6.02 N/µm), suggesting that the local contact response is influenced not only by the effective hardness of the coated surface system, but also by coating morphology and load transfer mechanisms. In Set II, contact stiffness ranged from 6.09 N/µm for specimen II.2 to 6.32 N/µm for specimen II.3, indicating a relatively uniform elastic response despite the differences observed in effective hardness of the coated surface system and deformation resistance. For Set III, the values increased from 6.17 N/µm for specimen III.2 to 6.68 N/µm for specimen III.3, the latter exhibiting the highest stiffness among all investigated coatings. This behavior is consistent with the elevated effective elastic modulus of the coated surface system and the partial contribution of the Ti6Al4V substrate during indentation. Comparing the three coating systems, Set III generally exhibited the highest contact stiffness values, followed by Sets I and II. Nevertheless, the relatively narrow range of variation (approximately 6.0–6.7 N/µm) indicates that contact stiffness is less sensitive to the coating composition than to the effective hardness of the coated surface system and the effective elastic modulus of the coated surface system, being primarily affected by the overall load-bearing capacity of the coating/substrate system and the degree of substrate involvement during indentation.

The influence of coating thickness and substrate support must be considered when interpreting the microindentation results. Because the indentation depth is not negligible relative to the thickness of several coatings, particularly the thinner layers, the deformation field may extend through the coating and interfacial region into the Ti6Al4V substrate. Therefore, the measured effective hardness of the coated surface system and the effective elastic modulus of the coated surface system represent effective properties of the coating–interface–substrate system, rather than the intrinsic properties of the phosphate coating alone. This influence is expected to become more pronounced as the coating thickness decreases or its structure becomes discontinuous. In such cases, the comparatively high hardness or effective elastic modulus of the coated surface system may reflect increased load support from the considerably stiffer Ti6Al4V substrate, rather than superior coating properties. This effect is particularly relevant for sample III.3, whose discontinuous morphology and substantial substrate exposure can explain the values approaching those of unlk uncoated Ti6Al4V. Conversely, thicker and more continuous coatings are expected to receive a comparatively greater contribution from the phosphate layer. Accordingly, comparisons among the formulations should be interpreted primarily in relation to their combined thickness, compactness, continuity, and deformation behavior. Lower-load nanoindentation or depth-dependent indentation measurements would be necessary in future studies to determine the comparative indentation response of the coatings while minimizing substrate influence.

The microindentation results represent the effective response of the combined coating–substrate system, rather than the intrinsic properties of the phosphate crystals. Because the indentation depth exceeded 10% of the coating thickness, the measured response was influenced by coating thickness, compactness, crystal morphology, structural continuity, and the underlying Ti6Al4V substrate. The available EDS point measurements do not demonstrate the existence or mechanical contribution of a chemically graded interfacial region.

### 3.3. Scratch Test

The scratch test results were therefore interpreted not only through average COF, friction force (F_f_) and AE values, but also through the progressive damage sequence Lc1–Lc3. For the present phosphate conversion coatings, Lc1 mainly corresponds to the cohesive damage of the crystalline phosphate layer, such as crystal fragmentation, compaction or microcracking. Lc2 corresponds to local adhesive damage, where SEM/EDX evidence indicates the partial exposure of the Ti6Al4V substrate. Lc3 corresponds to severe delamination or continuous coating removal. This distinction is important because phosphate conversion coatings may degrade by progressive crystal fracture and local wear without undergoing catastrophic interfacial delamination. The initial damage observed during scratching was predominantly cohesive, involving the fracture, crushing, and displacement of phosphate crystals within the scratch track. Local substrate exposure observed at higher loads may result from progressive coating penetration, abrasive removal, or localized adhesive failure; however, substrate exposure alone does not prove large-scale interfacial delamination. True adhesive failure was assigned only where post-test microscopy revealed coating separation or lateral delamination at the interface. No continuous or catastrophic delamination was observed for the better-performing coatings within the investigated load range.

Figure 4 show the graphs obtained from the scratch test for the experimental specimens I.1, II.3 and III.2. As shown, the COF for sample I.1 has an average of 0.54 and a standard deviation of 0.078; the coefficient of friction starts at 0.10 and increases to approximately 0.77 at an indenter travel distance of 3.5 mm, and subsequently, it decreases, reaching a relatively constant friction regime in the 7–10 mm range, with the COF fluctuating between 0.4 and 0.5—Figure 4a. Compared to Ti6Al4V, the COF value increases significantly, nearly doubling, which is explained by the surface roughness, the crystalline morphology of the layer, as well as the mechanical interaction between the crystals and the indenter [22,29]. At the same time, the graph shows that the F_f_ increases progressively, with an average value of approximately 2.73 N and a standard deviation of 1.43, reaching a maximum value of 4.9 N. This increase can be correlated with the increase in COF, and indicates the emergence of stronger friction mechanisms, such as the fragmentation of phosphate crystals, thus leading to the progressive deterioration of the layer. The acoustic emission for sample I.1 has an average of approximately 0.0027 V and a standard deviation of 0.0025 V. Throughout the test, the AE signal exhibits fluctuations indicating the presence of mechanical micro-events caused by crystal fracture, the local micro-delamination of the coating, or particle detachment. Compared to the AE signal of Ti6Al4V, the signal from sample I.1 indicates that more intense degradation mechanisms occur during the scratch test. The results are consistent with the values obtained from profilometric analysis and SEM observations; the surface characteristics of coating I.1 can lead to either crystal fracture or stress concentration at their tips. Regarding the microindentation results, they indicated the coating’s susceptibility to mechanical damage. COF exhibits strong fluctuations due to the initial roughness also observed by Surmeneva [30], where the first adhesive cracks appear at Lc = 5.3–5.6 N; at 7 N, no significant delamination occurs, only local detachments along the channel edge.

Under scratch loading, the phosphate coatings experience a complex stress state involving compression ahead of the stylus, shear stresses beneath the contact region and tensile stresses at the trailing edge. Consequently, the scratch response depends strongly on coating architecture. In coatings containing large isolated crystals and extensive intergranular regions, stress localization promotes crystal fracture and particle detachment, generating fluctuations in F_f_ and acoustic emission. In contrast, compact coatings composed of finer interconnected phosphate crystals distribute the contact stresses over a larger number of load-bearing interfaces, reducing local stress intensity and delaying crack propagation.

The improved scratch resistance of the more compact coatings may be attributed to their greater structural continuity, finer interconnected crystals, and more uniform distribution of contact stresses. The present measurements do not demonstrate any contribution from elemental diffusion or a mechanically graded interface.

Because the progressive-load scratch test was performed up to 10 N over a 10 mm track, the applied normal load increased approximately linearly at 1 N/mm. Therefore, the damage events observed on the COF, friction force and AE curves can be directly converted into critical-load intervals. For sample I.1, the first major COF increase and instability occurred around 3.5 mm, corresponding to Lc1 ≈ 3.5 N, which can be assigned to the onset of cohesive damage, mainly phosphate crystal fracture and local compaction. Since the COF subsequently stabilized between 7 and 10 mm and no catastrophic delamination was reported, adhesive failure was not reached within the applied range; therefore, Lc2 > 10 N and Lc3 > 10 N may be considered for this sample. For sample II.3, the high COF region between 1 and 3 mm indicates early interaction between the stylus and surface crystals, corresponding to Lc1 ≈ 1–3 N. The sudden decrease in COF and friction force observed between 7 and 9 mm corresponds to a load interval of Lc2 ≈ 7–9 N, suggesting local coating thinning, particle detachment or partial substrate exposure, but not continuous delamination. Thus, Lc3 was not reached up to 10 N. For sample III.2, the COF stabilized rapidly and the AE signal showed no major damage events, while SEM observations indicated no significant peeling or severe degradation; consequently, the coating did not show a clear cohesive or adhesive failure event within the tested range. Therefore, for III.2, both Lc1 and Lc2 may be considered higher than the maximum applied load or not clearly detected, while Lc3 > 10 N, indicating the highest scratch resistance and the best scratch-induced interfacial integrity among the investigated representative coatings. Combined with its compact morphology and favorable microindentation response, specimen III.2 exhibited the most advantageous overall tribological and mechanical behavior. This interpretation confirms that the coatings degrade mainly by progressive cohesive damage and local wear rather than catastrophic adhesive delamination. The critical loads reported here should be considered approximate values because they were derived from the combined interpretation of friction, acoustic emission and post-test SEM observations.

The tribological behavior of sample I.2 changed significantly, although the average COF is approximately 0.49 with a standard deviation of 0.16. This time, the test begins with high COF values, ranging from 0.8 to 1.05, reflecting the initial phase of contact between the indenter and the crystal tips. As the test proceeds, the COF decreases progressively, stabilizing at 0.35–0.45. This stabilization may be associated with the local smoothing of the crystals. The average friction force is 2.26 N, with a standard deviation of 0.68, and the maximum value (4.39 N) occurs near the end of the test, at approximately 9.5 mm. In all cases the F_f_ curve shows a progressive increase during the test. Local fluctuations in the F_f_ force may be associated with the micro-fracturing or detachment of phosphate crystals. The tribological contact is influenced by the layer’s morphology and compactness. The AE signal is approximately −0.002 V with a standard deviation of 0.002, exhibiting fluctuations similar to those of sample I.1, without major changes suggesting significative delamination of the layer, indicating that its degradation occurs gradually. The morphology and topographic analysis are consistent with the results, explaining the high initial COF value by the presence of the observed asperities and the high roughness. At the same time, the relatively low hardness of sample I.2 indicates that the layer is susceptible to deformation and local crystal fragmentation. Thus, based on these data, the layer degrades gradually, with areas where micro-fracturing of the crystals occurs.

For sample I.3, the behavior is similar to that of sample I.1. The COF has an average value of approximately 0.48 with a standard deviation of 0.13. The maximum value of COF is reached at approximately 1.5 mm. The rapid increase in COF can be associated with the initial contact between the indenter and phosphate crystals. After this, the COF values start to decrease, being stabilized around 5 mm. Close to the end of the test, around 7–8 mm, the COF value drops to 0.2, which can suggest a local delamination of the layer or the presence of a severe degradation mechanism. The F_f_, as in the other cases, evolves progressively, reaching a maximum of 4.16 and an average of 2.40, with a standard deviation of 0.87. The friction force graph presents a significant variation in the same with COF, which may be associated with crystal fragmentation and local detachment. The AE signal (−0.0008, standard deviation 0.002) exhibits relatively small fluctuations, with isolated pulses in certain areas, suggesting that the layer degradation is gradual. Although sample I.3 obtained the best values in the microindentation tests, the wear mechanism is more strongly influenced by the layer’s morphology. All three samples exhibit similar COF values, suggesting that the overall friction mechanism is similar. However, the way in which the coating degrades differs, with sample I.3 exhibiting the most stable friction behavior. At the same time, comparing F_f_ values shows that I.1 exhibits the highest friction resistance, while the other two samples exhibit lower values, suggesting more uniform contact.

Figure 4b shows the graph obtained from the scratch test performed on sample II.3. For II.1 sample, the COF decreases progressively throughout the test, starting from high values (up to 1.03) and stabilizing after 4.5–5 mm at values ranging from 0.45 to 0.50. Its average value is 0.57 with a standard deviation of 0.12. This behavior is due to the indenter’s initial contact with the crystals, and as it advances, the crystals are flattened or removed. At the same time, its fluctuations indicate the presence of microcracks in the crystals or their detachment, a phenomenon also observed in the samples from Set I. Regarding the F_f_, the graph shows a constant increase, reaching a maximum of 4.71 N, with an average of 2.78 N and a standard deviation of 0.96 N. This increase may be associated with the formation and accumulation of wear particles from broken crystals in the contact area, which can increase friction through abrasion. The AE signal exhibits moderate variations, with an average of approximately −0.001 and a standard deviation of 0.002, which may be associated with local processes such as crystal fracture or the rearrangement of surface contact. Thus, the tribological behavior of sample II.1 is characterized by an initial running-in phase, a gradual stabilization of the COF as the surface becomes uniform, a progressive increase in F_f_, and local microfracture processes or crystal detachment. These aspects are consistent with the morphology of the phosphate layer and the profilometric analysis, which contribute to both the high initial COF value and the tribological behavior. For the tribological behavior of sample II.2, as expected from the profilometric analysis results, the average COF value is the lowest among the samples studied, namely, 0.36, with a standard deviation of 0.13. However, it exhibits greater fluctuations compared to sample II.1, with values ranging from 0.07 to 0.72. The fact that the COF starts from low values is characteristic of surfaces with lower roughness, where initial friction is reduced; in this case, SEM observations indicate finer crystals and a more compact layer—Figures S4–S6. Furthermore, the microindentation tests showed that the layer exhibits higher hardness, with the crystals deforming less. At the same time, F_f_ has an average value of 1.92 with a standard deviation of 1.06. All of this is supported by the H/E and H^3^/E^2^ ratios, which indicate improved resistance to crack initiation and better stress distribution. In the 7–8 mm range, there is a sudden decrease in COF and F_f_, attributed to the formation of a layer of wear particles that temporarily acts as a solid lubricant; once this layer is removed, these values begin to rise again. This increase in the two parameters, with large fluctuations in values, may be due either to the more pronounced plastic deformation of the layer or to the accumulation of material in front of the indenter. The AE signal exhibits low fluctuations, without large spikes, having an average value of −0.00007 V with a standard deviation of 0.002. This suggests that no intense phenomena occurred during the test, but rather a controlled wear mechanism. Furthermore, it indicates that no delamination of the layer occurs in that area, but rather that progressive wear or local deformation occurs. As in the case of test II.1, for sample II.3, the specific COF graph is located in the 1–3 mm range at high values, fluctuating between 0.60 and 0.89, which indicates contact between the indenter and the crystals. Subsequently, it decreases gradually, reaching an average value of 0.54 with a standard deviation of 0.16. At the same time, the value of F_f_ increases progressively up to the 7–9 mm range, where a sudden decrease in both F_f_ and COF occurs. This area is likely influenced by the temporary formation of an intermediate layer of wear particles or by local changes in the friction mechanism. Toward the end of the test, the COF and F_f_ decrease again, likely due to wear particles accumulating and reducing friction. Throughout the test, the AE signal is relatively uniform, with an average of approximately −0.00016 V and a standard deviation of 0.002. This means that no major fractures or significant delamination of the layer occurred during the test. The fluctuations are associated with the detachment of wear particles, the microcracking of the crystals, and the rearrangement of surface contact. The behavior observed in the scratch test is consistent with the other results, where the roughness of the layer and the SEM images justify the high COF value.

The tribological behavior of specimen III.1, as determined by the scratch test, confirms that the COF fluctuations are quite large throughout the test, with an average value of 0.36 and a standard deviation of 0.112. In the 0–1 mm range, it increases rapidly, reaching a maximum value of 0.77, which may be correlated with the adaptation of the contact between the indenter and the crystal or with a possible removal of the crystal tips. After 1 mm, the COF gradually decreases but continues to exhibit high fluctuations, suggesting crystal compaction. After 5 mm, the COF becomes relatively constant, though its value still varies. Throughout the test, F_f_ increases, reaching a maximum of 3.22 N and an average of 1.79 N, with a standard deviation of 0.62 N. The AE signal has an average value of −0.0009 V with a standard deviation of 0.002. It is relatively constant, indicating no major phenomena except at the end of the test, where a significant variation occurs, likely due to a minor local detachment of the coating. The behavior suggests progressive coating wear, with local failure at 9.5 mm, consistent with the low hardness value. Figure 4c shows the results of the scratch test for sample III.2. The average COF in this case is significantly higher than that of sample III.1, with a value of 0.51 and a standard deviation of 0.08. Given the more uniform coating surface, contact between the indenter and the coating forms more quickly, leading to faster COF stabilization, as supported by the standard deviation. Throughout the test, the coating’s behavior is stable and continuous, with no significant crystal micro-fracturing or peeling. These observations are also supported by the slightly progressive increase in F_f_, with an average of 2.60 N and a standard deviation of 1.02, reaching a maximum of 4.43 at the end of the test, without causing significant degradation of the coating. Furthermore, the AE signal shows no major events, suggesting that the coating is holding up very well, with an average of −0.0003 V and a standard deviation of 0.002. Sample III.3 shows an average COF value of approximately 0.41 with a standard deviation of 0.1. The first part of the graph shown in Figure S11 indicates COF instability, which is attributed to the layer’s non-uniformity and discontinuity. At the same time, F_f_ increases irregularly, with sudden variations that can be attributed to local detachment or a temporary loss of contact between the indenter and the layer; its average value is 2.21 with a standard deviation of 0.98. After 2 mm, the AE signal exhibits very large spikes, indicating the local delamination of the coating or crystal breakage, with a mean of 0.007 and a standard deviation of 0.006. In this case, specimen III.3 exhibits unstable tribological behavior, which is explained by the progressive destruction of the coating and the exposure of the substrate. Thus, the scratch test results confirm that the coating is discontinuous, and are consistent with the results obtained by SEM.

### 3.4. SEM/EDX Analysis After Scratch Tests

The SEM images of sample I.1 (Figure 5a,b) reveal the scratch mark left by the indenter, which has a relatively smooth central area, while the edges show accumulations of material and crystal fragments, indicating that deformation of the layer and local fracturing of the phosphate crystals occurred. Analyses of sample I.2 show a more pronounced degradation mechanism—Figure S2. Thus, it can be observed that the wear mark is narrower than that of sample I.1, suggesting more localized degradation. Even though the fragmented crystals were removed or displaced toward the edge, they did not alter the indentation width. Compared to sample I.1, the indentation left by the indenter is not smooth; on the contrary, more prominent crystal fragments resulting from micro-fracturing can be observed on its surface. The edges of the wear mark are more irregular, with more pronounced fragmentation of the crystals around the channel. At higher magnification, cracks appear in the layer near the mark; they are particularly visible in areas with compact crystalline aggregates. Additionally, localized areas of dislocated and piled-up material appear as fine agglomerations along the edge. The specimen exhibits mixed behavior, with deformation by scratching and a more pronounced component of local layer rupture (micro-fracturing), suggesting lower internal cohesion than in specimen I.1. In the case of sample I.3, the size of the indentation is similar to that of sample I.2 and compared to the other two samples, the edges of the indentation no longer show high material accumulations, a feature indicating a more controlled degradation of the layer without extensive crystal fragmentation, even though the layer was progressively removed under the indenter, Figure S3. The presence of microcracks at the edge of the indentation suggests the occurrence of local stresses. However, the appearance of cracks did not lead to extensive delamination of the layer. In this case, the wear mechanism is dominated by the abrasion and local deformation of the crystalline structure.

These results are consistent with the evolution of the coefficient of friction, its fluctuations indicating processes of crystal fragmentation and the progressive exposure of the substrate. Thus, the results obtained indicate the partial deterioration of the phosphate layer under load. The tribological behavior of sample I.1 can be divided into stages. At the beginning of the test, the indenter comes into contact with the crystal tips, which are compressed and some are even deformed or cracked. Subsequently, the large crystals and intergranular zones become stress concentration points, leading to the formation of microcracks or fine fragments detached from the crystal, causing the layer to begin failing locally. In Figure 5c,d, for the surface of sample II.3, material build-up can be observed on the sides of the scratch mark left by the scratch test. As with the samples in Set I, the coating does not peel off; instead, the crystals deform or fragment and shift outward. No massive exfoliation occurs; however, there are signs of progressive damage to the layer (broken crystals, uneven edges). The layer withstands the mechanical stress of scratching through a combination of compaction and cracking; the relatively large width indicates a more modest resistance to tangential stress. The layer’s morphology explains the initially high COF, due to the large crystals. At the same time, it explains the decrease in the COF toward the end, which correlates with the fragmentation or deformation of the crystals as the surface becomes smoother. In the vicinity of the scratch, fine cracks and a transition zone where the layer appears compacted can be distinguished. Signs of crystal tip flattening following the scratch test, in which the debris is compacted and polished into a glaze layer, were also observed by Ernens [30], who further noted that zinc and zinc compounds are easier to remove than Mn-based compounds. In the case of sample II.2, the investigation shows a trace different from the one presented earlier. Thus, it was observed that there is greater material accumulation than in sample II.1, and in the central area of the trace, there are fewer traces of fractured or dislodged crystals; the deformation is predominantly plastic and uniformly distributed. The trace appears more uniform, with more stable edges and fewer abrupt fracture areas. Cracks are rarer and less extensive in the vicinity of the channel than in II.1 and II.3. Rather, controlled deformation and reduced material overlap at the edges are observed. In this case, the best local mechanical integrity of the layer in set II is observed (i.e., better resistance to crack propagation and spalling), consistent with the previous selection based on topographic criteria. The trace profile shows a narrower groove with higher edges due to the excess material in those areas. Therefore, the deformation appears more stable and homogeneous, in line with the scratch test results. Of all the samples analyzed so far, II.2 appears to have the highest resistance to delamination and wear.

At high magnification, for sample III.1, no delamination of the layer is observed; rather, plastic deformation of the crystals, specifically their fragmentation, is evident. The wear is progressive, as observed in the scratch test. Compared to the other specimens, the scratch mark profile is different, with deformed crystals present in the selected area. This difference can also be explained by the mechanism at work, namely, material accumulation and the compression of the crystals or their redistribution. Figure 5e,f shows representative images of the scratch mark obtained on the surface of specimen III.2. As can be seen, the size of the scratch mark is much smaller compared to that of specimen III.1, due to the mechanical properties of the coating, as determined by the microindentation test. In this case, the mark confirms the uniform plastic deformation of the layer, without delamination or crystal breakage, exhibiting better tribological behavior than that of sample III.1, making it the most balanced in set III. Sample III.3 exhibits an irregular scratch mark on its surface, where broken crystals and areas of material accumulation can be observed.

The EDX analysis for sample I.1 shown in Figure 6 reveals a change in the sample surface’s chemical composition in the area where the indenter passed. The increase in Ti percentage content compared to the EDX analysis of the sample before testing indicates that, in certain areas, the phosphate layer was partially removed, exposing the substrate. The chemical composition at the points highlighted in Figure 6, showing variations in the number of elements depending on the point’s location, confirm a decrease in the concentration of Zn, P, and O and an increase in Ti in the tested area. The detection of coating-associated elements at selected positions near the interface indicates local compositional overlap; however, it cannot establish diffusion, chemical bonding, or chemical anchoring. However, these observations should not be interpreted as an exact measurement of elemental diffusion or penetration depth. The results may also be affected by the EDS interaction volume, interface roughness, cross-sectional preparation, and the absence of precisely recorded distances between analysis points. Continuous line scans or elemental maps with calibrated distance measurements would be required to establish the compositional gradient and interfacial zone thickness more rigorously.

The presence of these fragments leads to the formation of abrasive particles that act as third bodies. As a result of these phenomena, the layer begins to degrade, thinning or being removed in certain areas, exposing the substrate. Thus, the mechanism at work is a combined one, driven by the micro-fracturing of the crystals, abrasion induced by detached particles, local delamination, and the progressive exposure of the substrate.

As with sample I.1, the EDX analysis for sample I.2 (Table 6) reveals significant increases in the concentrations of elements specific to the substrate and decreases in those specific to the phosphate layer. However, the presence of Zn, P, and O confirms that part of the layer remains present within the track. Compared to sample I.1, layer I.2 is removed to a greater extent, a finding supported by the Ti concentration at point 3 (72 wt.% for I.2 vs. 30.6 wt.% for I.1). As with the other layer, the tribological behavior can be divided into the same stages, but the severity of the phenomena is influenced by roughness as well as morphology.

EDX analysis showed that, although sample I.3 presents better mechanical properties than I.2, local degradation in some areas is more severe, with the substrate being the most exposed of the three samples (Table 7). When comparing all samples in Set I in terms of their chemical composition at various points, it can be seen that coating I.1 was the least removed of the three.

Table 8 presents the data obtained from the EDX analysis of the II.1 sample surface after the scratch test. As can be seen, the experiments were performed in the same way, in five points, similarly to the SEM images in Figure 6. In the central area, the substrate is most exposed (66.2 wt.%), and the elements specific to the phosphate layer (P, O, Zn) are present in the smallest quantities. Thus, the layer is not completely removed from the surface, but local wear is evident. Nevertheless, the absence of lateral delamination in the layer and the continued presence of its elements indicates that layer II.1 exhibits good scratch-induced interfacial integrity, with no evidence of continuous delamination up to 10 N applied to the substrate.

As shown in the EDX analysis (Table 9), in the case of sample II.2, the layer was not removed from the surface; rather, it underwent local deformation. In the scratch area, Ti appears in greater quantities, but the layer is not completely removed; rather, it is worn and thinned. These conclusions are consistent with the COF and F_f_ values. Compared to the other samples, in this case, there is no fragmentation of the crystals, but only their deformation and the progressive wear of the layer.

As observed in the previous tests, the layer of the sample II.3 is intermediate in terms of morphology and tribological behavior. This time, the mechanism is mixed, involving both deformation and crystal fragmentation. The intermediate behavior of sample II.3 indicates a balance between the layer’s stiffness and its deformation capacity.

As with the other specimens, the EDX analysis of sample II.3 (Figure 7) revealed a higher concentration of titanium in the indentation area, as well as the presence of elements such as P, O, and Zn, which confirms the mechanism and the fact that the coating was not completely removed following the test.

The results of the EDX analysis of the scratch mark are presented in Table 10. The values of elements specific to the substrate are present in large quantities in the area of the mark left by the indenter, while those specific to the coating are present but in smaller quantities. This finding confirms the presence of a progressive wear mechanism rather than the existing delamination of the coating.

From the EDX analysis results for III.2 given in Figure 8, it can be seen that points 2, 3, and 4 contain a high amount of Ti, but less than in sample III.1, which indicates that the layer remained in a greater quantity in sample III.2, although it is thinned or penetrated in certain areas by the indenter, confirming the tribological behavior observed in the scratch test. Sample III.2 appears to offer the best scratch resistance among the samples analyzed (high integrity, reduced damage, stable edges).

Table 11’s results confirm the conclusions drawn from the scratch test. The very high concentrations of substrate elements (Ti, V, and Al) at points 2, 3, and 4 confirm the nearly complete removal of the phosphate layer in III.3. This is also supported by the lower concentrations of the layer’s elements at the points in the scratch area compared to points 1 and 5. Of all the samples analyzed, this one has the lowest wear resistance. The scratch behavior observed for the phosphate conversion coatings deposited on Ti6Al4V is consistent with mechanisms previously reported for phosphate-based and bioactive coatings. Ernens et al. demonstrated that zinc and manganese phosphate coatings initially exhibit the brittle fracturing of their surface crystals, followed by the formation of a compacted tribological glaze layer generated through particle fragmentation and densification under sliding contact [30]. This transition from brittle fracture to compaction was identified as the dominant deformation mechanism rather than large-scale coating delamination [31]. Similarly, Toque et al. reported that the calcium phosphate coatings deposited by RF magnetron sputtering undergo progressive damage during scratch loading, including tensile cracking, trackside cracking, buckling and eventual delamination, highlighting the strong influence of coating microstructure and deposition conditions on failure evolution [32].

Ferraris et al. further demonstrated that the mechanical stability of bioactive surface layers is strongly dependent on the integrity of the interface, showing that scratch testing can effectively distinguish between surfaces characterized by localized damage and those exhibiting the extensive exposure of the underlying substrate [33]. In the case of phosphate conversion coatings on magnesium alloys, Liu et al. observed that laser surface texturing improved coating adhesion and significantly reduced coating shedding during scratch testing, indicating that surface morphology plays a crucial role in controlling failure mechanisms [34]. Likewise, Stuart et al. reported that phosphate-based glass coatings deposited on polished substrates failed through brittle tensile cracking and trackside delamination, whereas coatings deposited on roughened substrates exhibited substantially improved scratch resistance and reduced interfacial failure [35]. The present phosphate coatings displayed a damage mechanism that was dominated by gradual crystal fragmentation, local displacement and compaction of wear debris within the scratch track, with limited evidence of catastrophic delamination for the best-performing coatings. This behavior resembles the compaction-controlled response reported by Ernens et al., rather than the extensive interfacial exfoliation described by Toque et al. and Stuart et al., suggesting that the developed phosphate layers maintain sufficient interfacial integrity while accommodating scratch-induced stresses through localized particle rearrangement and densification.

The comparatively stable scratch response of the compact coatings may be attributed to their greater structural continuity, smaller intergranular regions, and more uniform distribution of contact stresses. The present EDS measurements do not establish a diffusion zone or a compositionally or mechanically graded interfacial region. Nevertheless, the present measurements do not establish a 15–20 μm diffusion zone or demonstrate that such a region compensates for lower coating hardness through stress redistribution. This mechanism remains a hypothesis that would require distance-calibrated compositional profiling and spatially resolved nanoindentation across the interface.

### 3.5. Profilometry Analysis

The profilometric analysis revealed parameter values that provide insights into the layer’s surface topography and scratch tracks. Thus, the average values of the three measurements for each sample, as well as the standard deviation, are presented in Table 12. Figure 9 shows the variations in the average values of R_a_ and R_p_. Analyzing all the values corresponded to Set I sample, it can be observed that I.2 presents the highest values for the determined parameters. Comparing the values of R_a_ and R_q_ for all three samples, a big difference between these values is seen for I.2, which suggest a topography with many intercrystalline zones and prominent crystals. In contrast, sample I.1 presents the smallest difference between them, indicating a more compact structure, all these observation being confirmed by SEM images. Regarding the R_p_ and R_v_, the lowest values are observed in sample I.3, indicating a finer, more uniform structure, consistent with the SEM images. The R_z_ values, as well as the discussions regarding the morphological analysis of the layers in set I, confirm that the surface of sample I.2 exhibits areas with pronounced profile maxima and minima, due to intergranular zones and polyhedral crystals, while I.1 and I.3 are characterized by relatively uniform layers, with a more compact structure and a finer structure, respectively. In all three cases, the R_sk_ value lies in the negative range, thus indicating a surface where minima predominate over maxima, which may be associated with the porous crystalline structure of the phosphate conversion layers. Moreover, comparing the R_sk_ values reveals that the values for I.1 and I.3 are close to 0, suggesting a relative uniform maxima distribution. The R_ku_ values of I.2 and I.3 samples exceed 3, suggesting an important difference between the dimension of crystals and intercrystalline areas. Meanwhile, sample I.1 exhibits a value less than 3, indicating less pronounced intergranular zones, with a smaller difference between the crystals and these zones. Another important aspect concerns the value of Rsm, which provides information on the uniform distribution of crystals and intergranular zones in samples I.1 and I.3 compared to I.2.

Based on the profilometric analysis presented, the Zn–Zr–Ca phosphate coating III.2 exhibited the most compact and homogeneous surface morphology, with the lowest roughness values among all investigated coatings (R_a_ = 0.79 ± 0.06 μm, R_q_ = 1.01 ± 0.11 μm, R_p_ = 1.82 ± 0.16 μm, and R_z_ = 3.84 ± 0.36 μm), while the Zn–Zr–Mg coating II.1 showed the highest roughness (R_a_ = 3.43 ± 0.25 μm and R_z_ = 14.14 ± 0.44 μm). These results are consistent with the SEM observations, which indicate a denser crystal packing and reduced intergranular porosity for sample III.2. When compared with data from the literature, the obtained roughness values fall within the range generally considered favorable for biomedical phosphate-based surfaces. Citeau et al. reported that calcium phosphate-treated Ti6Al4V surfaces with an average roughness of approximately 1.57 μm promoted osteoblastic activity and exhibited improved biological performance compared with smoother machined surfaces [36]. Similarly, Hayakawa et al. demonstrated that calcium phosphate-coated titanium implants with moderate roughness enhanced bone–implant contact and osseointegration [37]. Furthermore, Schwarz et al. showed that rougher calcium phosphate-modified titanium surfaces increased bone–implant contact, although excessively rough and heterogeneous topographies did not necessarily provide superior mechanical fixation [38]. In tribological applications, Podgornik et al. observed that phosphate coatings exhibiting controlled roughness and compact crystal morphology provided more stable friction behavior and improved resistance to galling [39]. Therefore, the low roughness and reduced profile height parameters measured for coating III.2, together with its homogeneous scratch track morphology and absence of significant delamination, suggest a favorable balance between tribological performance and potential biological response. These characteristics indicate that the Zn–Zr–Ca phosphate layer, particularly in sample III.2, may offer superior surface integrity and mechanical stability compared with the rougher phosphate coatings investigated in the present study.

The 3D images and topographic profile in Figure 9 highlight the scratch mark and the adjacent areas with more pronounced relief. Considering the scratch test results and these images, it can be concluded that the layer degradation results from the progressive fragmentation of crystals and their local removal from the scratch mark, with no severe delamination mechanisms observed. This indicates satisfactory adhesion but also a susceptibility to crystal breakage under applied force. Compared to sample I.1, the scratch mark profile on sample III.2 appears more pronounced, with sharper edges, confirming both the accumulation of material at the edges and a moderately uniform distribution. Even though F_f_ increased progressively, it exhibited significant variations at a certain point during the scratch test, suggesting crystal fragmentation.

Regarding Set II, sample II.1 exhibits a higher average roughness value, compared with Set I samples, consistent with observations under SEM. The R_sk_ value is situated in the positive range, but close to 0, which indicates a predominance of peaks that is in concordance with R_ku_ values, suggesting the presence of sharp asperities. Furthermore, the value of R_sm_ indicates the distance between sparse, large asperities, reflecting the well-developed crystals observed in SEM images—Figure 2. As for layer II.2, its degree of roughness is much lower, a finding confirmed by the SEM images. Compared to II.1, the value of R_sk_ is negative, indicating a surface dominated by valleys; at the same time, the values of R_sm_ and R_ku_ indicate that asperities are more frequent and more rounded, features also observed in the SEM. Among the samples in set II, layer II.3 has the smoothest surface, with the lowest R_a_ value. As in sample II.2, the surface is dominated by minima. However, the difference between the two layers lies in the value of R_ku_, which suggests the presence of sharper asperities and better-defined crystals in this case. Of all three samples, layer III appears to be the most uniform.

Figure 9 highlights the topographic profile of the scratch tracks and confirms the progressive wear mechanism of the phosphate conversion coatings. The profiles reveal that the degradation process is mainly governed by crystal fragmentation and localized material displacement rather than severe coating delamination. In the case of sample I.1, the scratch track presents moderate material accumulation at the edges, indicating the gradual deformation of the lamellar phosphate crystals under mechanical loading. Sample II.3 exhibits a more pronounced groove and sharper profile variations, suggesting higher local stress concentration and crystal fragmentation during sliding. By contrast, sample III.2 shows a narrower and more uniform scratch profile, which demonstrates improved load distribution, enhanced coating compactness, and superior resistance to crack propagation and wear. Overall, the results in Figure 9 confirm that the tribological response of the coatings is strongly dependent on their microstructural uniformity and crystal morphology, with the more compact and homogeneous layers exhibiting better mechanical integrity and scratch resistance. The comparatively favorable scratch response of selected coatings may be associated with their observed compactness, continuity, crystal connectivity, and reduced intergranular spacing. However, the present results do not demonstrate that elemental diffusion, chemical anchoring, or a mechanically graded interface is responsible for improved stress transfer or resistance to delamination. Establishing such relationships would require correlated chemical and mechanical characterization across the interface.

The profilometric analysis of the samples in set III has revealed the roughness of the layers. Thus, III.1 exhibits the highest values of R_a_ and R_q_, consistent with the crystalline morphology of the layer observed by SEM. In addition, it exhibits the highest values of the parameters R_v_, R_p_, and R_z_, confirming the presence of intergranular zones and well-defined crystals. The moderate compactness of the layer is also supported by the negative value of R_sk_, which indicates the presence of intergranular zones of significant sizes. At the same time, the value of R_sm_ supports the SEM observations, as it is associated with larger crystals [40,41]. Of the three samples in set III, as observed in SEM, sample III.2 exhibits the highest degree of compactness, confirmed by the low values of R_a_ and R_q_. Furthermore, this is supported by the values of R_v_, R_p_, and R_z_, which indicate a surface with minimal height variations, suggesting a uniform distribution of the crystals. The value of R_sk_ for sample III.2 is very close to 0, indicating that the layer surface is nearly symmetrical topographically [42]. All of this is supported by the low R_sm_ value, which suggests a compact, fine microstructure. Following the profilometric analysis of layer III.3, some interesting values were obtained. Thus, sample III.3 exhibits intermediate roughness and a more balanced profile than sample III.1. Furthermore, the positive value of R_sk_ indicates that the analyzed area is dominated by peaks, likely associated with crystal agglomerations rather than actual layer compactness.

Of the three sets, the samples in Set III (III.2 and III.3) exhibit the lowest average roughness and maximum profile peak height. At the same time, the roughest surface is that of sample II.1. Thus, it can be observed that the surface topography of the layers varies depending on the chemical composition of the phosphating solution, as well as on the amount of metal ions added to the solution, which subsequently influences all other properties of the material.

Although the present results demonstrate that coating chemistry and microstructure strongly influence surface roughness, micromechanical response, scratch resistance, and coating integrity, these findings should not be interpreted as demonstrating complete biomedical performance. Corrosion resistance and electrochemical stability were not evaluated in physiologically relevant media, and the possible release of Zn, Mg, Ca, Zr, or other species from the coatings was not quantified. Furthermore, no biological tests were performed to assess cytocompatibility, cell adhesion and proliferation, inflammatory response, hemocompatibility, or antibacterial activity. Consequently, the designation “bioactive” refers here to the incorporation of elements commonly associated with potentially beneficial biological functions, rather than to the biological activity demonstrated experimentally in this study. Future work should combine immersion and electrochemical testing in simulated physiological environments with ion release measurements and appropriate in vitro biological assays. Longer-term degradation, wear–corrosion interactions, coating stability under cyclic loading, and, subsequently, in vivo performance should also be investigated before the suitability of these coatings for biomedical implantation can be established.

## 4. Conclusions

Nine phosphate conversion coatings based on Zn–Mg–Ca, Zn–Zr–Mg, and Zn–Zr–Ca systems were successfully deposited on Ti6Al4V by chemical conversion processing at 90 °C. The phosphating bath chemistry strongly influenced crystal growth and the resulting coating architecture, producing significant differences in coating morphology, compactness, and thickness. SEM and EDS analyses confirmed the formation of phosphate-rich surface layers, and revealed local compositional differences among the coating, near-interface, and substrate regions. However, the discrete EDS point measurements did not establish a continuous elemental gradient, diffusion depth, or chemically graded interfacial zone.

The microstructural evolution of the coatings was governed by the balance between crystal nucleation and growth. Zn-rich formulations promoted the formation of larger phosphate crystals and thicker coatings, whereas the incorporation of Mg, Ca and Zr modified crystal growth behavior and increased nucleation density. As a consequence, coatings evolved from heterogeneous structures with large intergranular regions toward more compact architectures characterized by finer crystal assemblies and improved structural uniformity.

Microindentation testing demonstrated that the mechanical response depended primarily on coating architecture and interfacial load transfer, rather than on coating thickness alone. Because the indentation depth was substantial relative to the coating thickness, the measured hardness and effective elastic modulus characterize the combined response of the phosphate coating and Ti6Al4V substrate. Among the investigated coatings, specimen III.2 exhibited the most favorable mechanical performance, combining high compactness, low contact depth, elevated H/E and H^3^/E^2^ ratios, and improved resistance to elastic and plastic deformation. Specimen II.2 also demonstrated a balanced mechanical response associated with its refined and homogeneous microstructure.

Scratch testing revealed that coating degradation occurred predominantly through progressive crystal fragmentation, local compaction and limited wear, rather than catastrophic interfacial delamination. The superior scratch response of sample III.2 was associated with its compact and comparatively homogeneous coating architecture. The present measurements do not establish whether elemental diffusion or a mechanically graded interface contributed to load transfer between the coating and substrate.

Overall, the results demonstrate a direct relationship between phosphating chemistry, coating architecture and tribological performance. Among the investigated systems, the Zn–Zr–Ca formulation corresponding to specimen III.2 provided the most favorable combination of morphology, micromechanical behavior and scratch resistance, making it the best-performing coating within the investigated experimental domain.

The present study was limited to morphological, compositional, micromechanical and tribological characterization. Although the coatings contain biologically relevant elements such as Zn, Mg, Ca and Zr, no electrochemical, ion-release or biological investigations were performed. Consequently, no conclusions regarding corrosion behavior in physiological environments, biocompatibility, osseointegration or clinical performance can be drawn, and these aspects should be addressed in future studies.

## Figures and Tables

**Figure 1:**
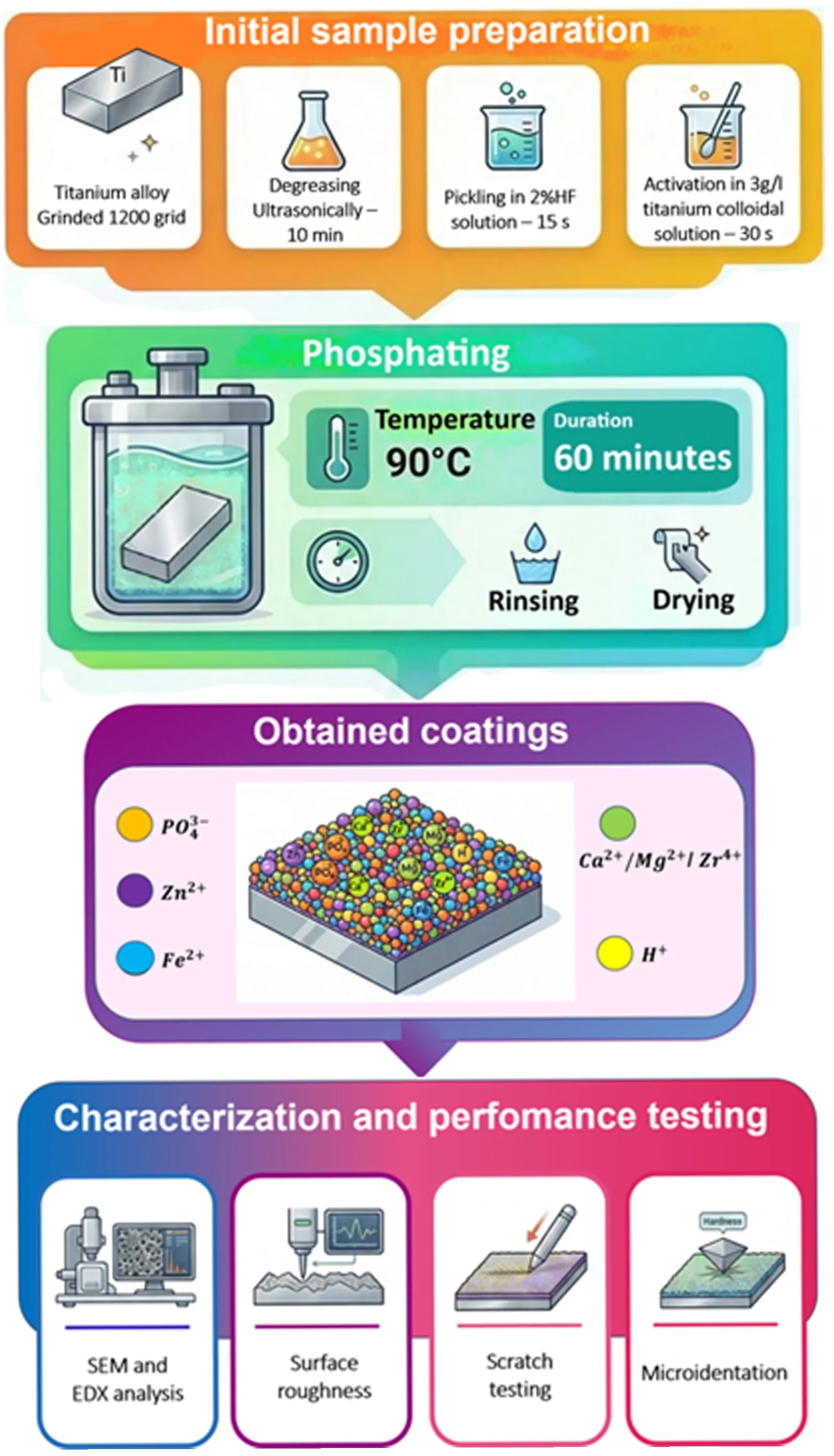
Schematic representation of the experimental workflow, including Ti6Al4V substrate preparation, chemical activation, phosphating treatment at 90 °C for 60 min, rinsing and drying, followed by SEM–EDX characterization, surface roughness measurements, scratch testing, and instrumented microindentation.

**Figure 2:**
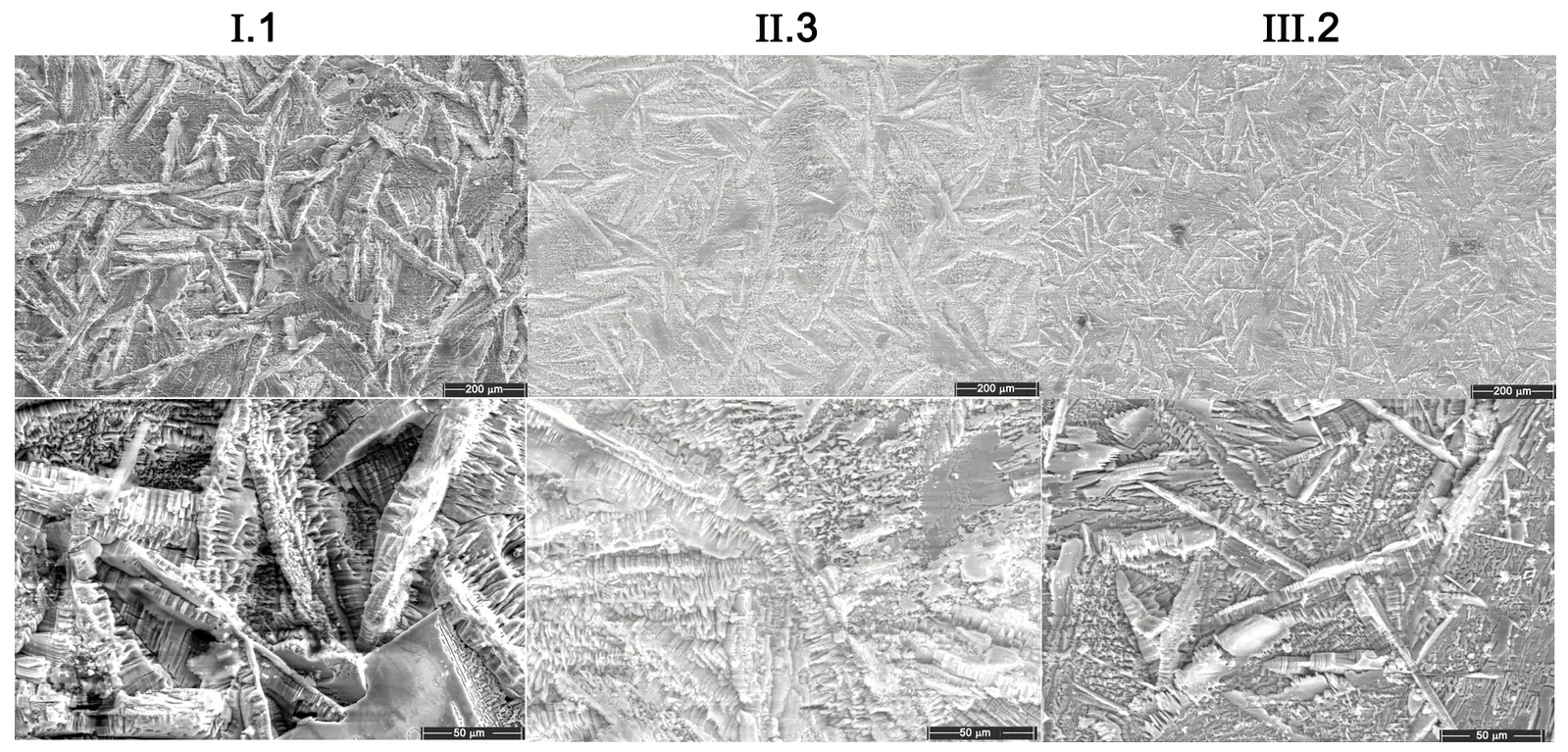
SEM micrographs of representative phosphate layers.

**Figure 3:**
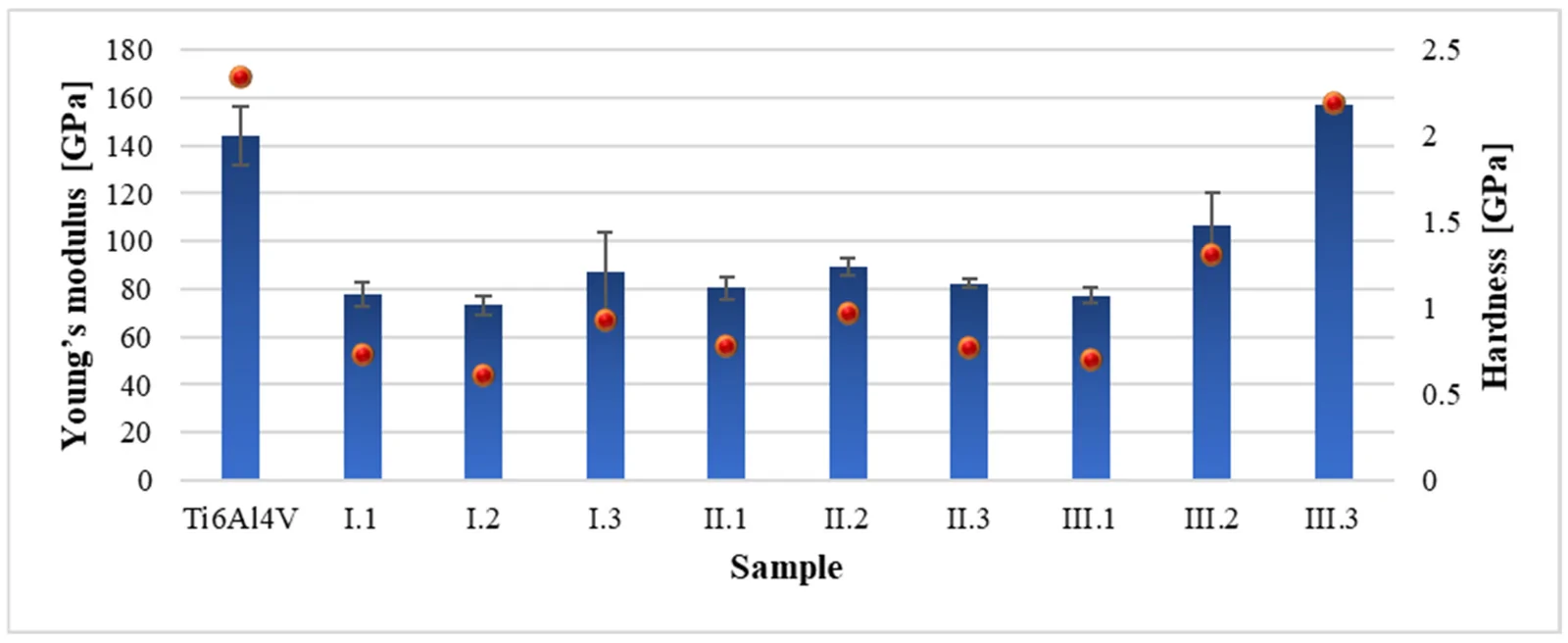
The H and E values of the studied samples compared with those of Ti6Al4V.

**Figure 4:**
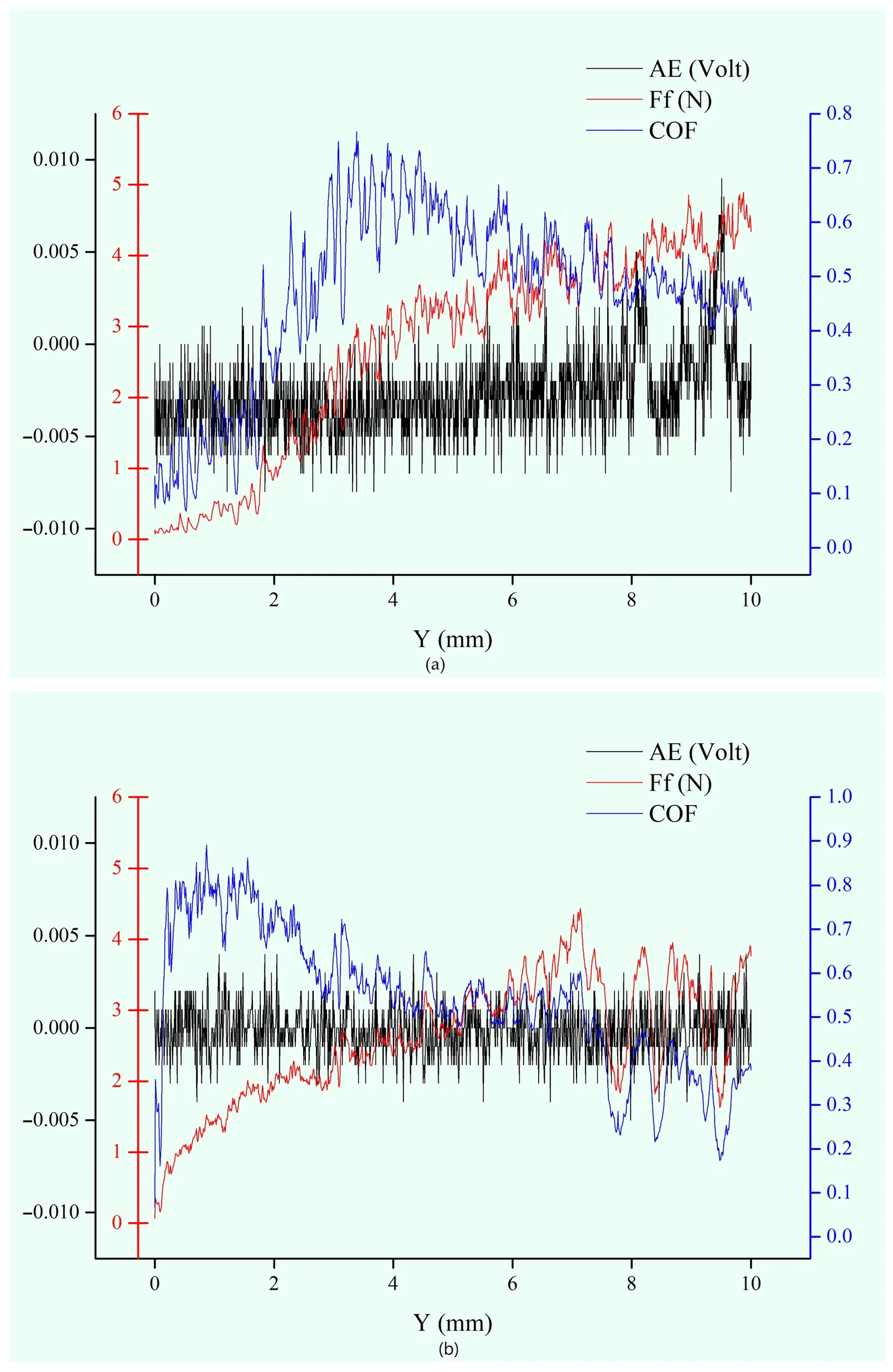

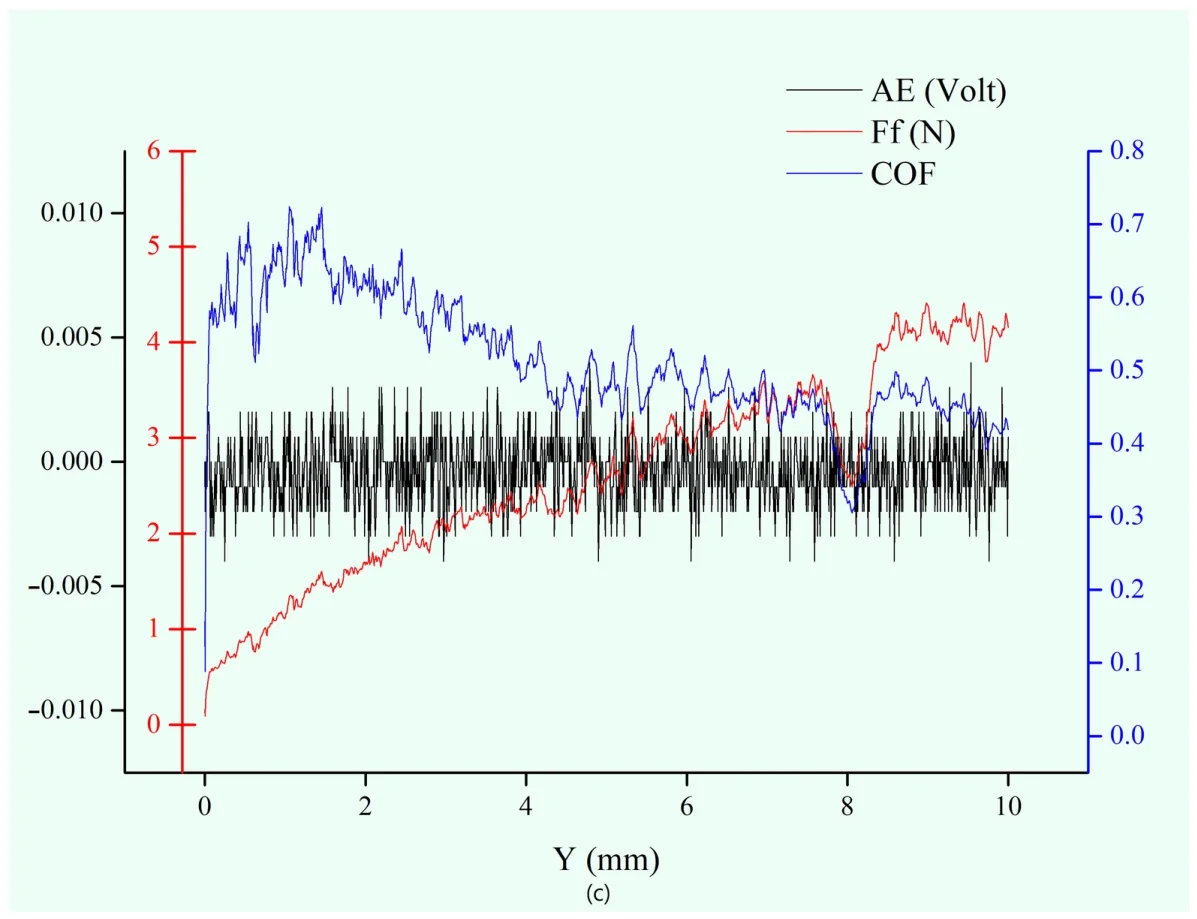
Representative scratch test curves for coatings (**a**) I.1, (**b**) II.3 and (**c**) III.2.

**Figure 5:**
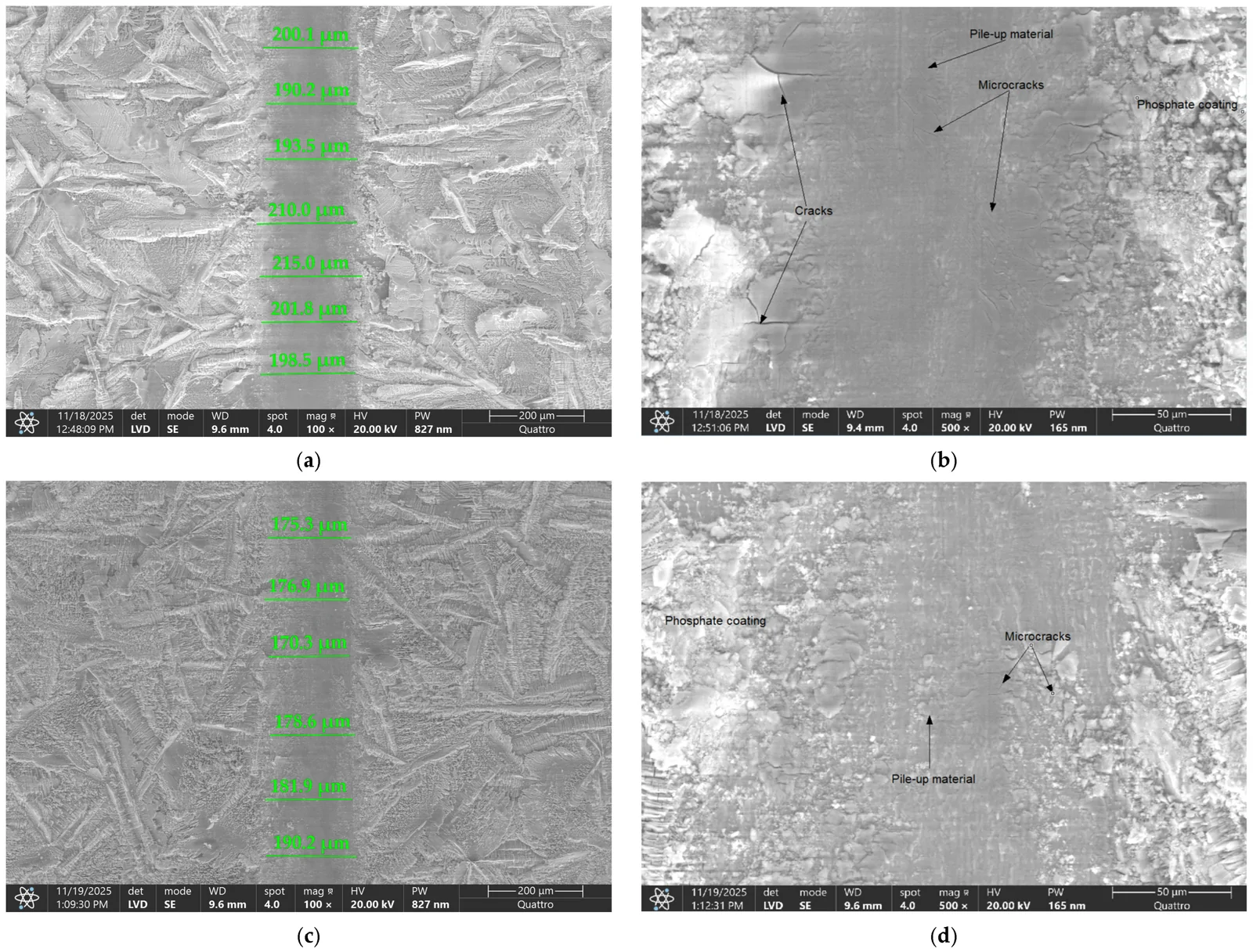

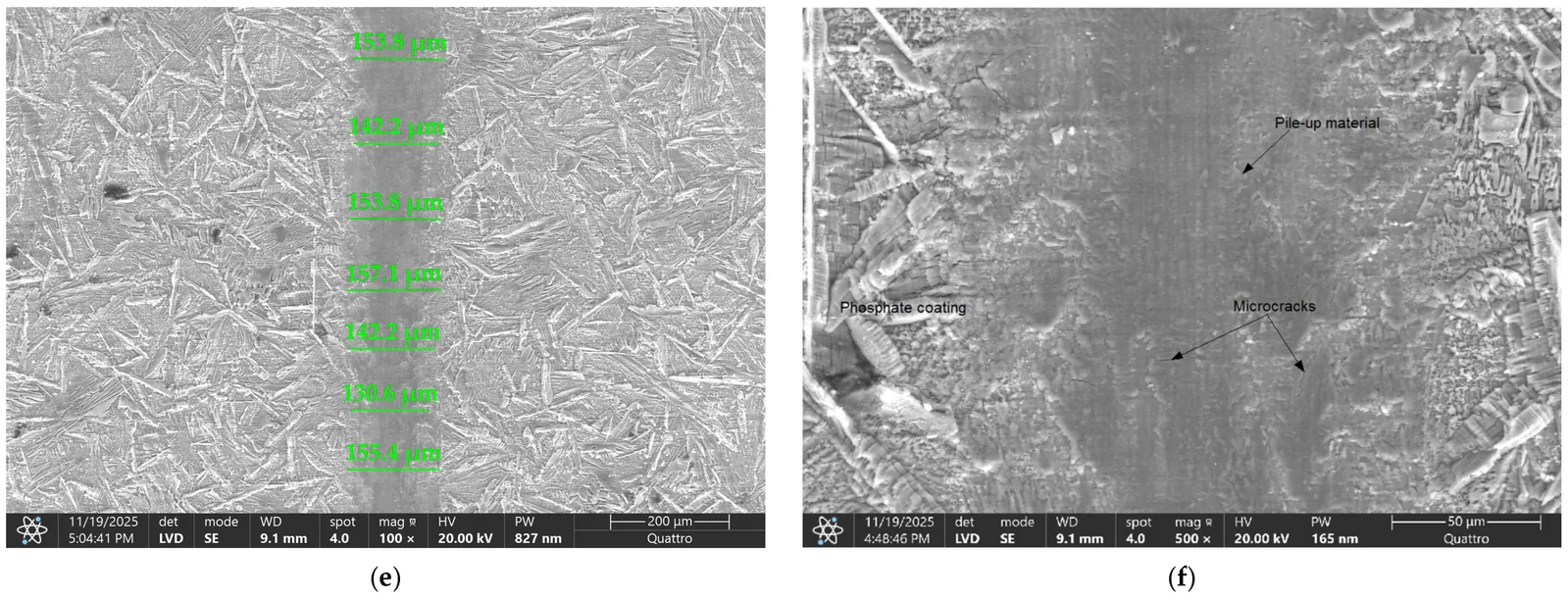
SEM images of the scratched surfaces: (**a**) SEM micrograph of scratch track profile 100×; (**b**) SEM micrograph at 500× of I.1, (**c**,**d**) II.3 and (**e**,**f**) III.2.

**Figure 6:**
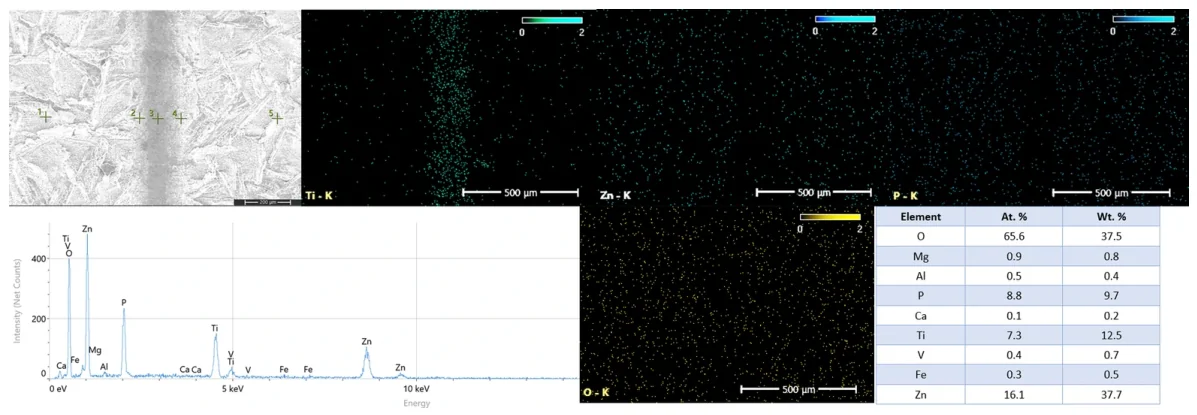
SEM image of the scratch track on sample I.1 showing the EDX analysis positions, corresponding elemental distribution maps, representative EDX spectrum, and area analysis results.

**Figure 7:**
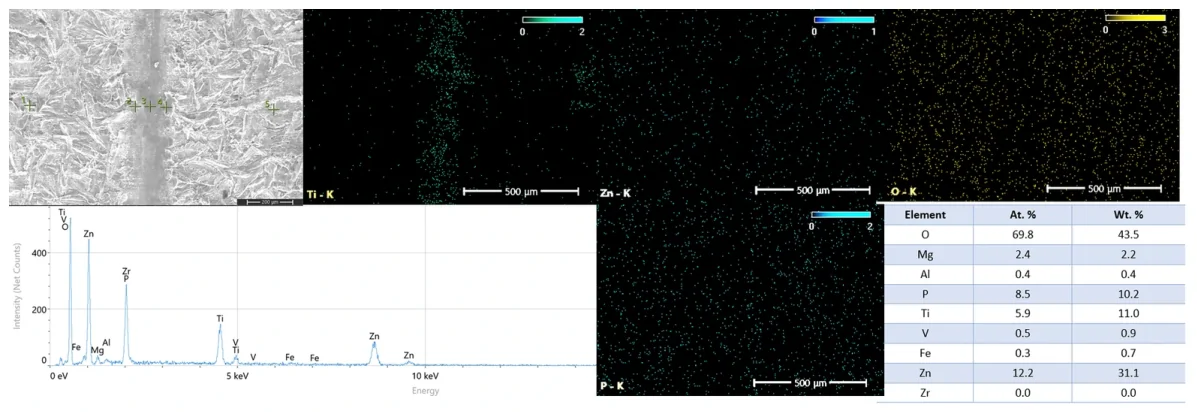
SEM image of the scratch track on sample II.3 showing the EDX analysis positions, corresponding elemental distribution maps, representative EDX spectrum, and area analysis results.

**Figure 8:**
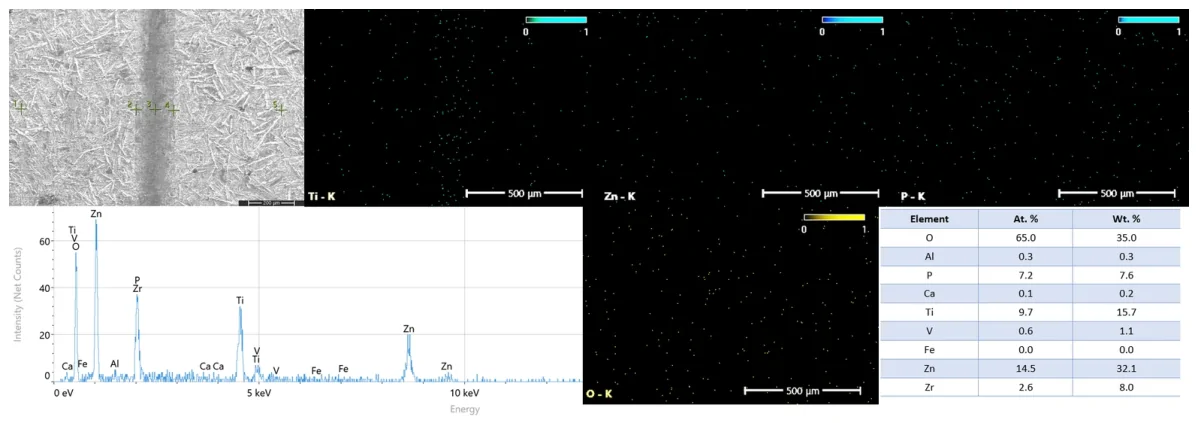
SEM image of the scratch track on sample III.2 showing the EDX analysis positions, corresponding elemental distribution maps, representative EDX spectrum, and area analysis results.

**Figure 9:**
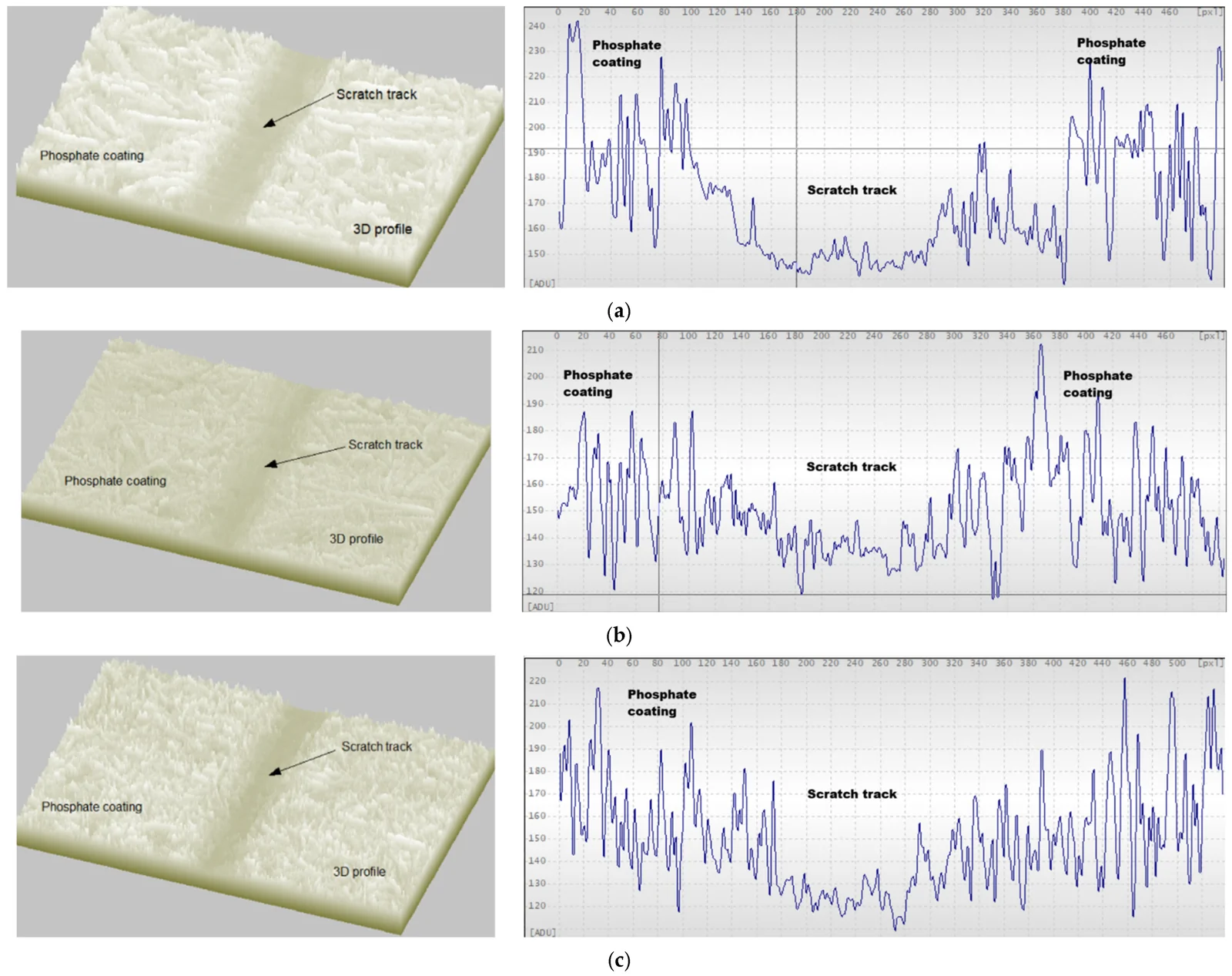
VegaTC intensity-based pseudo-3D visualization and corresponding grayscale intensity profile extracted from secondary electron SEM images of the scratch tracks: (**a**) I.1, (**b**) II.3, and (**c**) III.2. These representations are qualitative and should not be interpreted as calibrated surface topography or depth measurements.

**Table 1:** Chemical compositions of phosphating solutions.

Substance	I.1	I.2	I.3	II.1	II.2	II.3	III.1	III.2	III.3
	Quantity								
NaNO_2_ (g)	0.25	0.25	0.25	0.25	0.25	0.25	0.25	0.25	0.25
Na_5_P_3_O_10_ (g)	0.05	0.05	0.05	0.05	0.05	0.05	0.05	0.05	0.05
NaF (g)	0.42	0.42	0.42	0.42	0.42	0.42	0.42	0.42	0.42
H_3_PO_4_ (ml)	12.00	14.00	8.00	12.00	12.00	13.00	12.00	9.00	8.00
HNO_3_ (ml)	6.00	4.00	4.00	6.00	4.00	4.00	6.00	4.00	4.00
MgCO_3_ (g)	1.00	4.00	2.0	2.00	5.00	2.00	−	−	−
Ca(NO_3_)_2_ · 4H_2_O (g)	2.00	2.00	7.50	−	−	−	1.50	2.00	7.50
Zn (g)	4.00	1.50	1.50	2.50	1.50	1.50	4.00	1.50	1.50
ZrO_2_ (g)	−	−	−	3.00	2.00	5.00	2.00	5.00	2.00
Double-distilled water	Rest	Rest	Rest	Rest	Rest	Rest	Rest	Rest	Rest
Fe (g)—for 24 h and after removed	5.00	5.00	5.00	5.00	5.00	5.00	5.00	5.00	5.00

A 5.00 g piece of metallic Fe was immersed in each freshly prepared phosphating solution for 24 h and subsequently removed before phosphating the Ti6Al4V specimens.

**Table 2:** Chemical composition of the surface coatings (average values from three different areas of 1 mm^2^).

Element	I.1		II.3		III.2	
	wt.%	at.%	wt.%	at.%	wt.%	at.%
Oxygen (O)	35.4	64.1	38.6	66.8	38.2	67.1
Magnesium (Mg)	0.8	1.0	1.4	1.6	-	-
Aluminum (Al)	0.1	0.1	0.0	0.0	10.0	9.1
Phosphorus (P)	12.8	12.0	11.8	10.6	0.2	0.2
Calcium (Ca)	0.1	0.0	-	-	0.0	0.0
Titanium (Ti)	2.0	1.2	4.1	2.4	10.8	6.3
Vanadium (V)	0.0	0.0	0.5	0.3	0.8	0.4
Iron (Fe)	0.9	0.4	1.0	0.5	0.5	0.2
Zinc (Zn)	47.9	21.2	41.2	17.4	37.2	16.0
Zirconium (Zr)	-	-	1.4	0.4	2.3	0.7

St.Dev.: O: ±2; Mg: ±0.1; Al: ±0.01; P: ±0.1; Ca: ±0.01; Ti: ±0.1; V: ±0.1; Fe: ±0.01; Zn: ±1.2; Zr: ±0.1.

**Table 3:** The average of microindentation parameters for Zn–Mg–Ca samples.

Parameter	I.1	I.2	I.3
Maximum Load [N]	9.02 ± 0.01	9.01 ± 0.01	9.01 ± 0.01
Maximum displacement (h_max_) [µm]	11.28 ± 1.20	13.22 ± 1.07	9.24 ± 1.71
Young’s modulus (E) [GPa]	77.60 ± 5.14	73.15 ± 3.89	86.91 ± 16.89
Reduced modulus [GPa]	81.49 ± 5.07	77.14 ± 3.84	90.35 ± 16.17
Hardness (H) [GPa]	0.73 ± 0.09	0.61 ± 0.05	0.93 ± 0.18
Contact stiffness [N/µm]	6.14 ± 0.15	6.34 ± 0.06	6.02 ± 0.48
Contact depth [µm]	10.17 ± 1.22	12.15 ± 1.07	8.11 ± 1.61
Elastic strain index (H/E)	0.009 ± 0.0006	0.008 ± 0.0002	0.010 ± 0.00007
Plastic deformation index (H^3^/E^2^) [GPa]	6.54 × 10^−5^ ± 1.64 × 10^−5^	4.08 × 10^−5^ ± 4.29 × 10^−6^	1.04 × 10^−4^ ± 2.16 × 10^−5^

**Table 4:** The average of microindentation parameters for Zn–Zr–Mg samples.

Parameter	II.1	II.2	II.3
Maximum Load [N]	8.99 ± 0.01	9.01 ± 0.01	9.01 ± 0.01
Maximum displacement (h_max_) [µm]	10.58 ± 0.74	8.81 ± 1.32	10.62 ± 0.16
Young’s modulus (E) [GPa]	80.43 ± 4.92	89.29 ± 3.86	82.44 ± 1.80
Reduced modulus [GPa]	84.25 ± 4.80	92.78 ± 3.69	86.20 ± 1.74
Hardness (H) [GPa]	0.78 ± 0.05	0.97 ± 0.15	0.77 ± 0.01
Contact stiffness [N/µm]	6.14 ± 0.13	6.09 ± 0.34	6.32 ± 0.08
Contact depth [µm]	9.48 ± 0.72	7.69 ± 1.33	9.55 ± 0.14
Elastic strain index (H/E)	0.009 ± 0.0001	0.011 ± 0.0014	0.009 ± 0.00009
Plastic deformation index (H^3^/E^2^) [GPa]	7.23 × 10^−5^ ± 6.57 × 10^−6^	1.17 × 10^−4^ ± 4.4 × 10^−5^	6.71 × 10^−5^ ± 3.0 × 10^−7^

**Table 5:** The average of microindentation parameters for Zn–Zr–Ca samples.

Parameter	III.1	III.2	III.3
Maximum Load [N]	9.01 ± 0.01	9.01 ± 0.01	9.01 ± 0.01
Maximum displacement (h_max_) [µm]	11.64 ± 0.64	6.93 ± 1.52	4.33 ± 0.32
Young’s modulus (E) [GPa]	77.29 ± 3.16	106.62 ± 14.07	156.95 ± 2.48
Reduced modulus [GPa]	81.19 ± 3.08	108.98 ± 13.05	153.68 ± 2.10
Hardness (H) [GPa]	0.70 ± 0.04	1.31 ± 0.35	2.19 ± 0.22
Contact stiffness [N/µm]	6.25 ± 0.29	6,17 ± 0.34	6.68 ± 0.35
Contact depth [µm]	10.56 ± 0.67	5.83 ± 1.56	3.32 ± 0.37
Elastic strain index (H/E)	0.009 ± 0.0006	0.012 ± 0.0019	0.014 ± 0.0014
Plastic deformation index (H^3^/E^2^) [GPa]	5.77 × 10^−5^ ± 1.17 × 10^−6^	2.08 × 10^−4^ ± 1.1 × 10^−4^	4.37 × 10^−4^ ± 1.24 × 10^−4^

**Table 6:** Chemical composition of the I.2 coating.

Element	Point 1		Point 2		Point 3		Point 4		Point 5	
	wt.%	at.%	wt.%	at.%	wt.%	at.%	wt.%	at.%	wt.%	at.%
Oxygen (O)	22.5	50.0	44.7	70.5	14.6	33.0	30.9	56.9	42.4	69.2
Magnesium (Mg)	1.5	2.2	1.2	1.3	0.2	0.3	0.7	0.9	2.1	2.3
Aluminum (Al)	0.7	0.9	0.7	0.7	4.8	6.5	1.8	2.0	0.0	0.0
Phosphorus (P)	7.5	8.6	5.5	4.5	0.9	1.0	2.3	2.2	12.8	10.8
Calcium (Ca)	0.3	0.2	0.0	0.0	0.0	0.0	0.0	0.0	0.2	0.1
Titanium (Ti)	6.4	4.7	29.5	15.6	71.8	54.6	52.6	32.4	3.1	1.7
Vanadium (V)	0.6	0.4	2.2	1.1	2.7	1.9	2.6	1.5	0.4	0.2
Iron (Fe)	2.0	1.3	0.2	0.1	0.4	0.2	0.2	0.1	1.2	0.6
Zinc (Zn)	58.5	31.7	16.0	6.2	4.6	2.5	8.9	4.0	37.8	15.1

**Table 7:** Chemical composition of the I.3 coating.

Element	Point 1		Point 2		Point 3		Point 4		Point 5	
	wt.%	at.%	wt.%	at.%	wt.%	at.%	wt.%	at.%	wt.%	at.%
Oxygen (O)	26.7	55.0	18.0	39.0	6.9	17.7	13.4	30.9	39.1	66.5
Magnesium (Mg)	0.7	0.9	0.1	0.1	0.0	0.0	0.3	0.4	1.9	2.1
Aluminum (Al)	0.6	0.8	4.1	5.3	5.1	7.8	4.4	6.0	0.1	0.1
Phosphorus (P)	9.1	9.7	1.6	1.8	0.9	1.2	1.5	1.8	12.7	11.2
Calcium (Ca)	0.7	0.6	0.1	0.1	0.1	0.1	0.1	0.1	0.3	0.2
Titanium (Ti)	7.5	5.2	67.8	48.9	79.6	67.9	70.7	54.5	5.2	2.9
Vanadium (V)	0.6	0.4	2.9	1.9	3.9	3.1	4.8	3.5	0.5	0.3
Iron (Fe)	0.8	0.5	0.6	0.4	0.2	0.1	0.4	0.3	0.1	0.0
Zinc (Zn)	53.3	26.9	4.8	2.5	3.3	2.1	4.4	2.5	40.1	16.7

**Table 8:** Chemical composition of the II.1 coating.

Element	Point 1		Point 2		Point 3		Point 4		Point 5	
	wt.%	at.%	wt.%	at.%	wt.%	at.%	wt.%	at.%	wt.%	at.%
Oxygen (O)	21.1	48.4	34.7	62.2	19.1	40.9	40.5	68.1	38.7	67.1
Magnesium (Mg)	0.2	0.4	1.3	1.6	0.3	0.4	0.7	0.8	1.0	1.2
Aluminum (Al)	0.3	0.4	0.4	0.4	3.7	4.7	0.2	0.2	0.0	0.0
Phosphorus (P)	9.9	11.7	13.5	12.5	1.0	1.2	12.5	10.9	12.8	11.5
Titanium (Ti)	3.0	2.3	6.8	4.1	66.2	47.4	6.2	3.5	1.4	0.8
Vanadium (V)	0.0	0.0	0.1	0.1	2.3	1.5	0.5	0.3	0.2	0.1
Iron (Fe)	0.6	0.4	0.9	0.5	0.4	0.3	0.7	0.3	0.6	0.3
Zinc (Zn)	64.9	36.4	42.2	18.6	6.0	3.2	37.8	15.6	44.5	18.8
Zirconium (Zr)	0.0	0.0	0.1	0.0	1.0	0.4	0.9	0.3	0.8	0.2

**Table 9:** Chemical composition of the II.2 coating.

Element	Point 1		Point 2		Point 3		Point 4		Point 5	
	wt.%	at.%	wt.%	at.%	wt.%	at.%	wt.%	at.%	wt.%	at.%
Oxygen (O)	36.7	63.9	30.7	56.6	39.3	66.0	43.5	69.4	36.0	62.6
Magnesium (Mg)	2.5	2.9	0.4	0.5	1.4	1.5	2.6	2.7	0.9	1.0
Aluminum (Al)	0.4	0.5	2.9	3.1	0.7	0.7	0.1	0.1	0.6	0.6
Phosphorus (P)	14.2	12.8	2.0	2.0	6.2	5.4	14.6	12.0	4.2	3.8
Titanium (Ti)	1.2	0.7	54.4	33.5	30.7	17.2	4.7	2.5	42.8	25.0
Vanadium (V)	0.2	0.1	1.7	1.0	1.8	1.0	0.0	0.0	2.4	1.3
Iron (Fe)	1.1	0.6	0.0	0.0	0.4	0.2	0.6	0.3	0.7	0.4
Zinc (Zn)	42.9	18.2	6.0	2.7	19.5	8.0	32.3	12.6	12.4	5.3
Zirconium (Zr)	0.8	0.3	1.9	0.6	0.0	0.0	1.6	0.4	0.0	0.0

**Table 10:** Chemical composition of the III.1 coating.

Element	Point 1		Point 2		Point 3		Point 4		Point 5	
	wt.%	at.%	wt.%	at.%	wt.%	at.%	wt.%	at.%	wt.%	at.%
Oxygen (O)	49.5	74.9	25.5	51.3	31.0	57.3	8.0	20.3	39.2	67.5
Calcium (Ca)	0.0	0.0	0.4	0.3	0.2	0.1	0.0	0.0	0.4	0.3
Aluminum (Al)	1.1	1.0	1.1	1.2	2.5	2.8	4.8	2.8	0.3	0.3
Phosphorus (P)	10.5	8.2	10.5	1.9	1.8	1.8	0.9	1.8	11.1	9.9
Titanium (Ti)	11.1	5.6	57.3	38.4	53.0	32.8	78.1	65.9	8.2	4.7
Vanadium (V)	0.6	0.3	3.0	1.9	2.3	1.3	2.6	2.1	0.7	0.4
Iron (Fe)	0.3	0.1	0.0	0.0	0.0	0.0	0.0	0.0	0.4	0.2
Zinc (Zn)	26.9	9.9	9.1	4.4	7.5	3.4	5.2	3.2	39.3	16.6
Zirconium (Zr)	0.0	0.0	1.8	0.6	1.7	0.5	0.4	0.2	0.4	0.1

**Table 11:** Chemical composition of the III.3 coating.

Element	Point 1		Point 2		Point 3		Point 4		Point 5	
	wt.%	at.%	wt.%	at.%	wt.%	at.%	wt.%	at.%	wt.%	at.%
Oxygen (O)	42.1	69.3	8.8	22.0	3.5	9.6	3.1	8.4	35.0	63.3
Calcium (Ca)	0.4	0.3	0.1	0.1	0.0	0.0	0.0	0.0	0.3	0.2
Aluminum (Al)	0.7	0.7	4.9	7.2	5.4	8.6	5.7	9.2	1.1	1.2
Phosphorus (P)	11.4	9.7	0.5	0.6	0.6	0.9	0.7	1.0	11.6	10.8
Titanium (Ti)	10.9	6.0	77.9	64.8	84.3	76.5	81.9	75.3	9.2	5.6
Vanadium (V)	0.8	0.4	4.2	3.3	2.5	2.1	3.5	3.0	0.6	0.3
Iron (Fe)	0.5	0.2	0.5	0.3	0.0	0.0	0.1	0.1	0.3	0.1
Zinc (Zn)	33.2	13.4	2.1	1.3	2.9	1.9	3.5	2.3	40.6	18.1
Zirconium (Zr)	0.0	0.0	1.0	0.4	0.8	0.4	1.5	0.7	1.3	0.4

**Table 12:** The average value of the parameters determined by profilometric analysis.

Roughness Parameter	I.1	I.2	I.3	II.1	II.2	II.3	III.1	III.2	III.3
Average roughness (R_a_), µm	2.31 ± 0.05	2.95 ± 0.04	2.13 ± 0.05	3.43 ± 0.25	1.60 ± 0.50	1.21 ± 0.04	2.15 ± 0.14	0.79 ± 0.06	1.05 ± 0.08
Root mean square roughness (R_q_), µm	2.85 ± 0.08	3.78 ± 0.09	2.75 ± 0.05	4.42 ± 0.12	1.98 ± 0.60	1.56 ± 0.05	2.90 ± 0.14	1.01 ± 0.11	1.35 ± 0.11
Maximum profile peak height (R_p_), µm	4.44 ± 0.07	5.41 ± 0.11	4.28 ± 0.07	6.94 ± 0.14	3.22 ± 0.90	2.62 ± 0.05	4.24 ± 0.13	1.82 ± 0.16	2.40 ± 0.16
Maximum profile valley depth (R_v_), µm	5.66 ± 0.4	7.93 ± 0.40	5.52 ± 0.34	7.30 ± 0.16	3.93 ± 1.12	3.50 ± 0.43	5.62 ± 0.15	2.02 ± 0.20	2.38 ± 0.19
Maximum height of the profile (R_z_), µm	10.16 ± 0.25	13.34 ± 0.50	9.80 ± 0.41	14.14 ± 0.44	7.15 ± 2.02	5.75 ± 0.15	9.86 ± 0.28	3.84 ± 0.36	4.78 ± 0.35
Skewness of the profile (R_sk_)	−0.24 ± 0.23	−0.54 ± 0.06	−0.26 ± 0.07	0.25 ± 0.15	−0.10 ± 0.004	−0.12 ± 0.02	−0.33 ± 0.03	−0.05 ± 0.13	0.16 ± 0.11
Kurtosis of the profile (R_ku_)	2.75 ± 0.02	3.60 ± 0.35	3.52 ± 0.02	3.46 ± 0.85	2.88 ± 0.09	3.55 ± 0.15	3.44 ± 0.62	3.24 ± 0.34	3.75 ± 0.05
Mean width of the profile elements (R_sm_), µm	135.6 ± 14.2	153.9 ± 19.5	137.01 ± 0.43	214.91 ± 14.68	117.98 ± 8.32	132.45 ± 7.16	139.56 ± 7.09	96.84 ± 9.50	132.45 ± 6.93

## Data Availability

The original contributions presented in this study are included in the article/Supplementary Materials. Further inquiries can be directed to the corresponding author.

## Supplementary Materials

The following supporting information can be downloaded at https://www.mdpi.com/article/10.3390/ma19194058/s1, Figure S1. Cross-sectional SEM images and EDS point-analysis positions for sample I.1. Points 1–3 are located within the phosphate coating, while points 4–6 extend from the coating–substrate interface into the Ti6Al4V substrate. The total distance covered by the point-analysis sequence is 14.22 μm. Table S1. Atomic percentages results on sample I.1. Figure S2. SEM images of sample I.2 (transversal view) + coating. Table S2. Atomic percentages results on sample I.2. Figure S3. SEM images of sample I.3 (transversal view) + coating. Table S3. Atomic percentages results on sample I.3. Figure S4. SEM images of sample II.1 (transversal view) + coating. Table S4. Atomic percentages results on sample II.1. Figure S5. SEM images of sample II.2 (transversal view) + coating. Table S5. Atomic percentages results on sample II.2. Figure S6. SEM images of sample II.3 (transversal view) + coating. Table S6. Atomic percentages results on sample II.3. Figure S7. SEM images of sample III.1 (transversal view) + coating. Table S7. Atomic percentages results on sample III.1. Figure S8. SEM images of sample III.2 (transversal view). Table S8. Atomic percentages results on sample III.2. Figure S9. SEM images of sample III.3 (transversal view). Table S9. Atomic percentages results on sample III.3. Figure S10. Indentation graphs for the phosphated samples. Figure S11. Variations in the coefficient of friction (COF) over the 10-mm distance on which the scratch resistance tests were performed (with a constant or linear load of 10 N) (a) sample I.1, (b) I.2, (c) I.3, (d) II.1, (e) II.2, (f) II.3, (g) III.1, (h) III.2, and (i) III.3. Figure S12. The I.2 sample scratch plot. Figure S13. The I.3 sample scratch plot. Figure S14. The II.1 sample scratch plot. Figure S15. The II.2 sample scratch plot. Figure S16. The III.1 sample scratch plot. Figure S17. The III.3 sample scratch plot.
