# Supplementary material for: Microstructure, Adhesion and Tribomechanical Performance of Zn–Mg–Ca- and Zn–Zr-Based Phosphate Conversion Coatings on Ti6Al4V Alloy
Source: Materials (Basel). 2026 Sep 23;19(19):4058. doi: 10.3390/ma19194058 (PMC13665734; doi:10.3390/ma19194058)
Supplement: Supplementary file 1 [file materials-19-04058-s001.zip › materials-4552030-supplementary.pdf]

Supplementary Files of the paper: MICROSTRUCTURE, ADHESION AND TRIBOMECHANICAL PERFORMANCE OF BIOACTIVE Zn–Mg–Ca- AND Zn–Zr-BASED PHOSPHATE CONVERSION COATINGS ON MEDICAL-GRADE TI6AL4V ALLOY

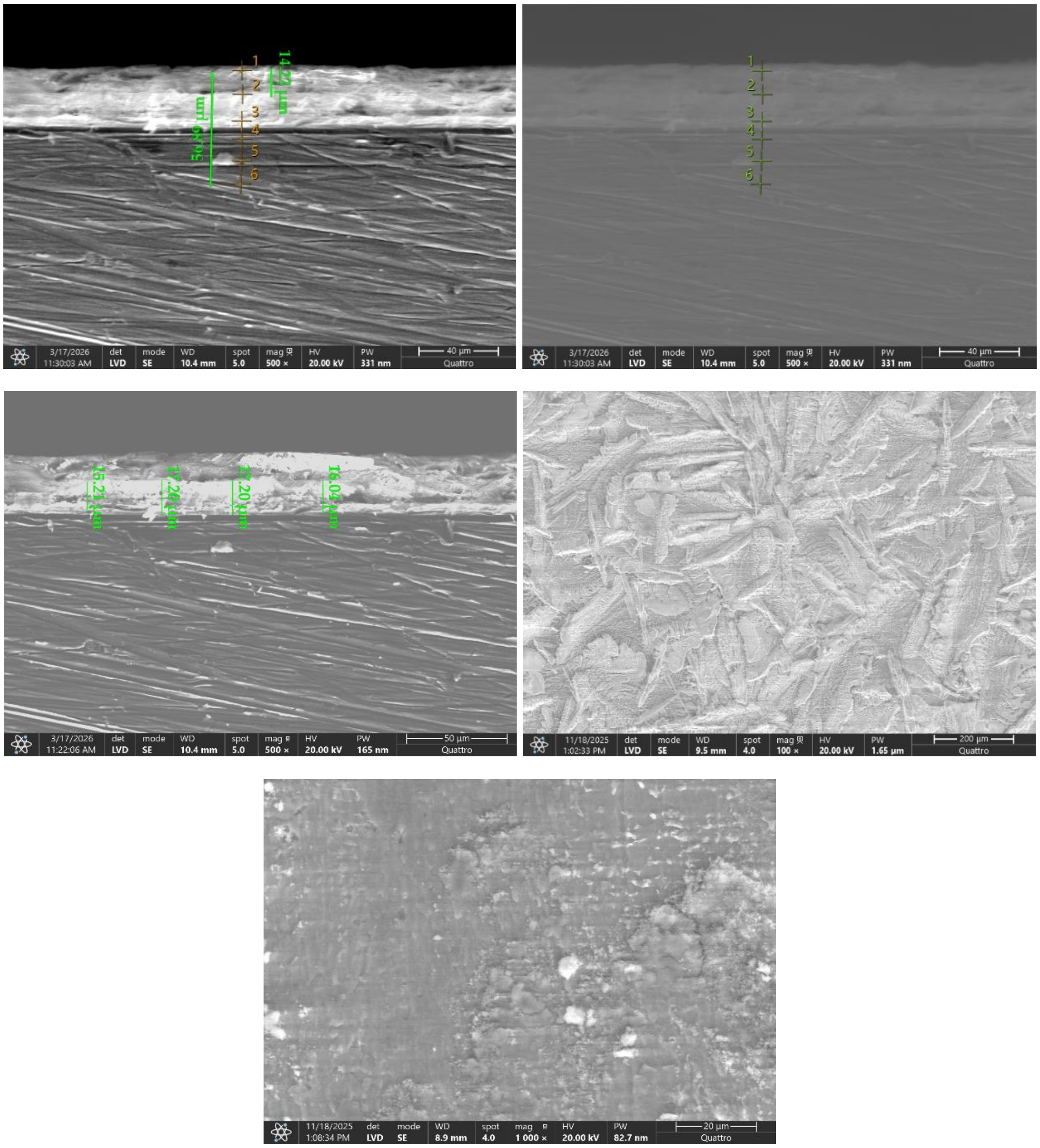

Figure S1 Cross-sectional SEM images and EDS point-analysis positions for sample I.1. Points 1–3 are located within the phosphate coating, while points 4–6 extend from the coating–substrate interface into the Ti6Al4V substrate. The total distance covered by the point-analysis sequence is 14.22 µm.

The EDS point-analysis values reported in Tables S1–S9 correspond to individual local measurements at the indicated positions. Because replicate measurements were not performed at each point, standard deviations are not reported for these data.

Table S1. Atomic percentage results for sample I.1 .

	Atomic %					
**Element**	**Point 1**	**Point 2**	**Point 3**	**Point 4**	**Point 5**	**Point 6**
O	70.3	69.6	65.8	0.0	0.0	0.0
Mg	0.4	0.5	0.3	0.0	0.0	0.0
Al	2.3	3.2	3.9	11.2	12.1	10.7
P	8.8	8.1	4.8	0.5	1.0	0.3
Ca	0.1	0.1	0.1	0.0	0.1	0.0
Ti	6.7	7.4	16.9	84.7	83.4	85.3
V	0.3	0.3	0.8	2.6	2.6	3.2
Fe	0.2	0.2	0.1	0.2	0.1	0.1
Zn	10.9	10.6	7.3	0.8	0.7	0.4

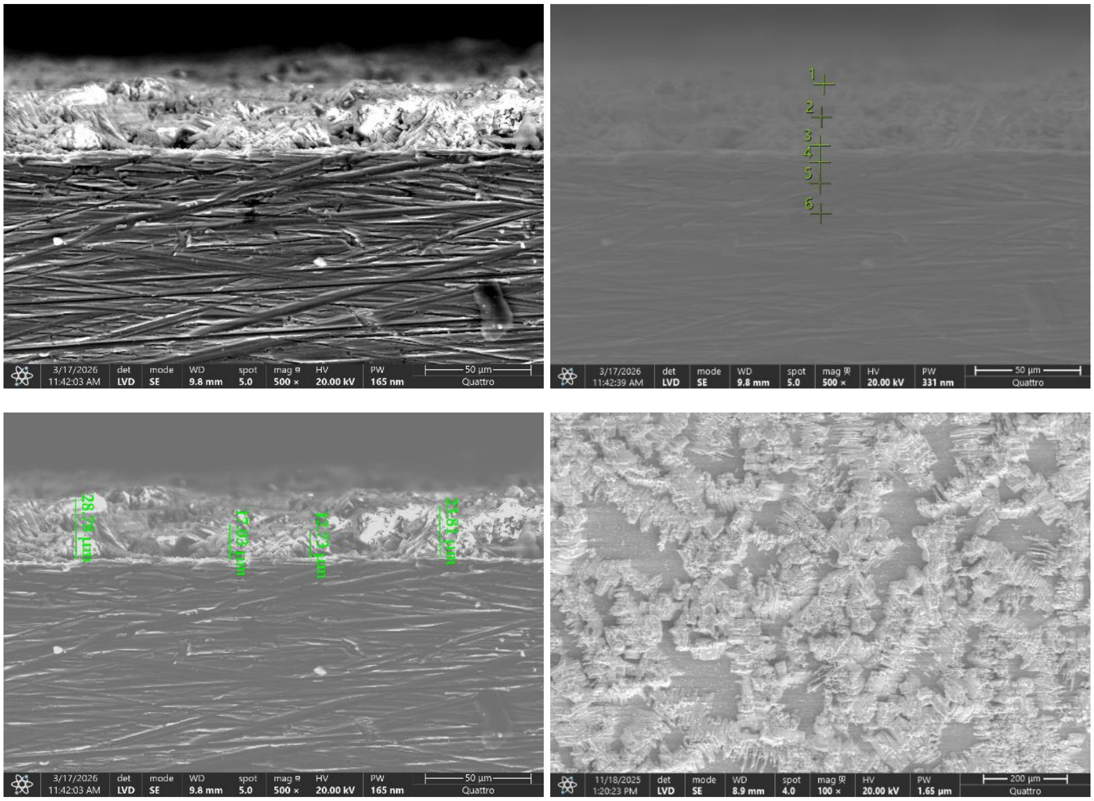

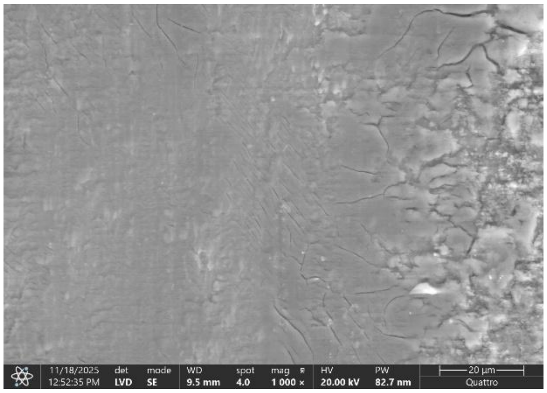

Figure S2. SEM images of sample I.2 (transversal view) + coating.

Table S2. Atomic percentage results for sample I.2.

	Atomic %					
**Element**	**Point 1**	**Point 2**	**Point 3**	**Point 4**	**Point 5**	**Point 6**
O	57.1	63.4	68.2	11.2	0.0	0.0
Mg	1.1	1.6	1.5	0.4	0.2	0.1
Al	20.9	12.3	11.4	20.8	21.7	20.5
P	6.3	7.3	5.7	0.8	0.6	0.4
Ca	0.2	0.1	0.1	0.0	0.0	0.1
Ti	6.4	6.1	6.5	63.5	74.0	74.3
V	0.3	0.3	0.3	1.9	2.5	3.6
Fe	0.5	0.4	0.1	0.2	0.2	0.3
Zn	7.2	8.5	6.2	1.2	0.8	0.7

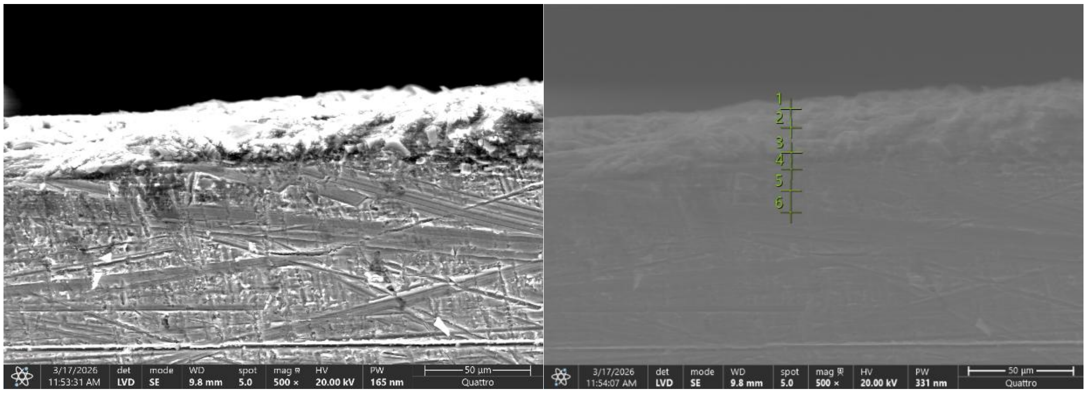

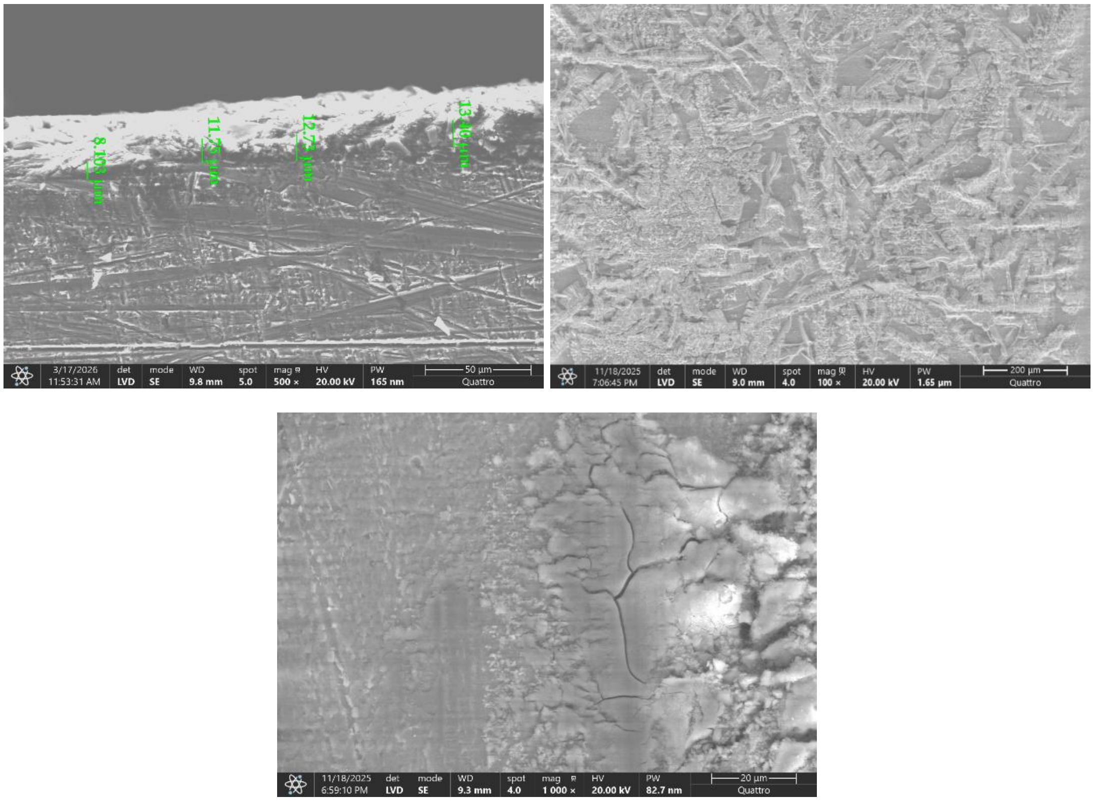

Figure S3. SEM images of sample I.3 (transversal view) + coating.

Table S3. Atomic percentage results for sample I.3.

	Atomic %					
**Element**	**Point 1**	**Point 2**	**Point 3**	**Point 4**	**Point 5**	**Point 6**
O	58.0	60.2	62.5	47.5	45.8	0.0
Mg	0.9	0.8	1.1	0.4	0.6	0.2
Al	23.1	14.6	12.2	12.9	12.4	22.7
P	6.1	4.9	7.6	2.1	2.8	0.6
Ca	0.2	0.2	0.5	0.4	0.1	0.0
Ti	4.8	12.4	7.3	32.4	31.6	73.4
V	0.2	0.6	0.3	1.2	1.9	2.2
Fe	0.1	0.1	0.1	0.1	0.1	0.2
Zn	6.6	6.2	8.4	3.0	4.7	0.7

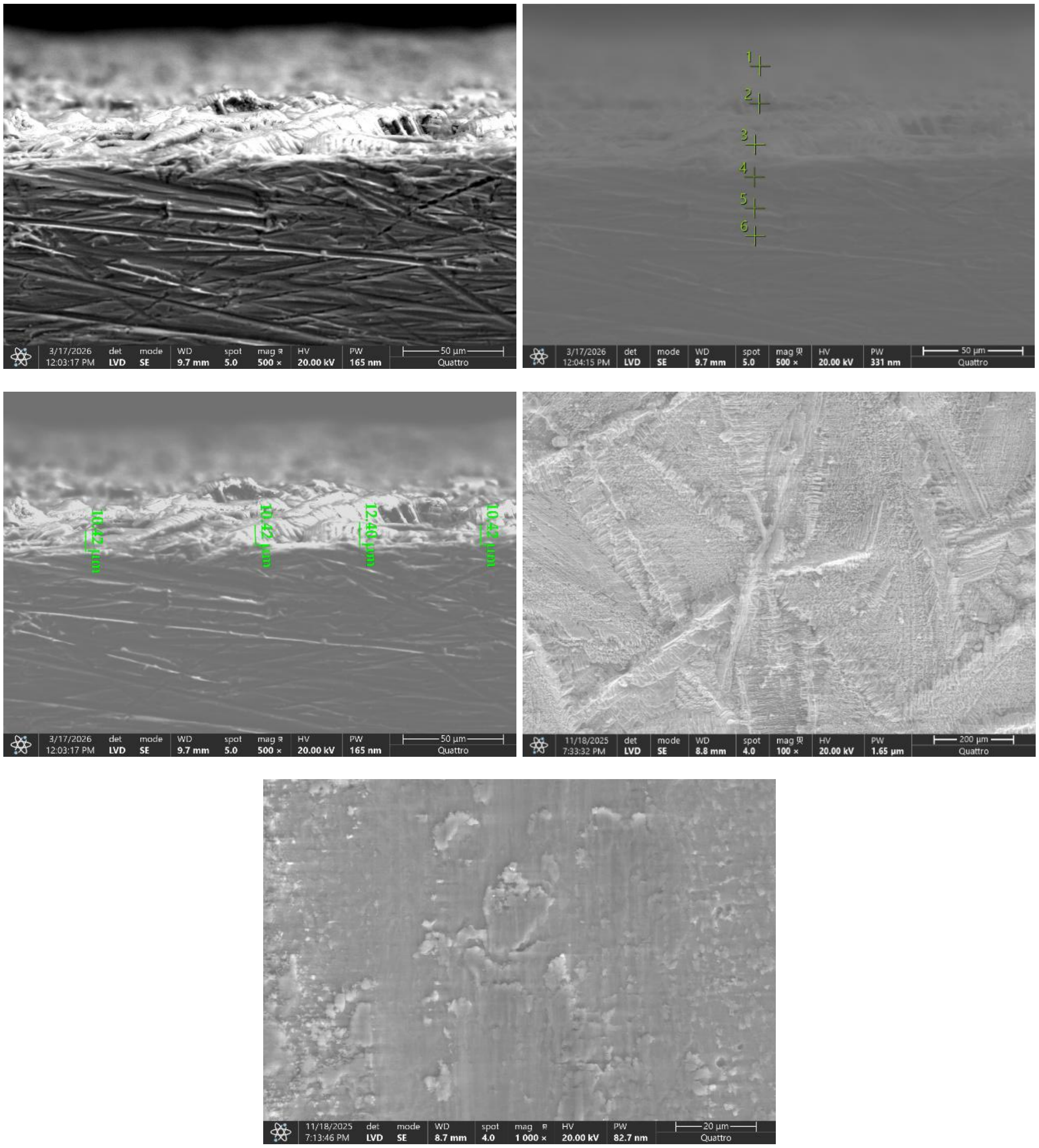

Figure S4. SEM images of sample II.1 (transversal view) + coating.

Table S4. Atomic percentage results for sample II.1.

	Atomic %					
**Element**	**Point 1**	**Point 2**	**Point 3**	**Point 4**	**Point 5**	**Point 6**
O	45.0	16.1	36.5	0.0	0.0	0.0
Mg	0.1	0.0	0.1	0.0	0.0	0.0
Al	13.2	10.4	15.4	8.7	7.6	8.5
P	0.8	0.4	1.0	0.0	0.0	0.0
Ti	37.7	69.6	34.6	88.1	88.5	87.7
V	1.4	2.5	1.4	2.9	3.4	3.4
Fe	0.3	0.4	0.2	0.1	0.2	0.2

	Atomic %					
**Element**	**Point 1**	**Point 2**	**Point 3**	**Point 4**	**Point 5**	**Point 6**
Zn	1.1	0.5	10.3	0.1	0.1	0.0
Zr	0.4	0.1	0.5	0.1	0.2	0.2

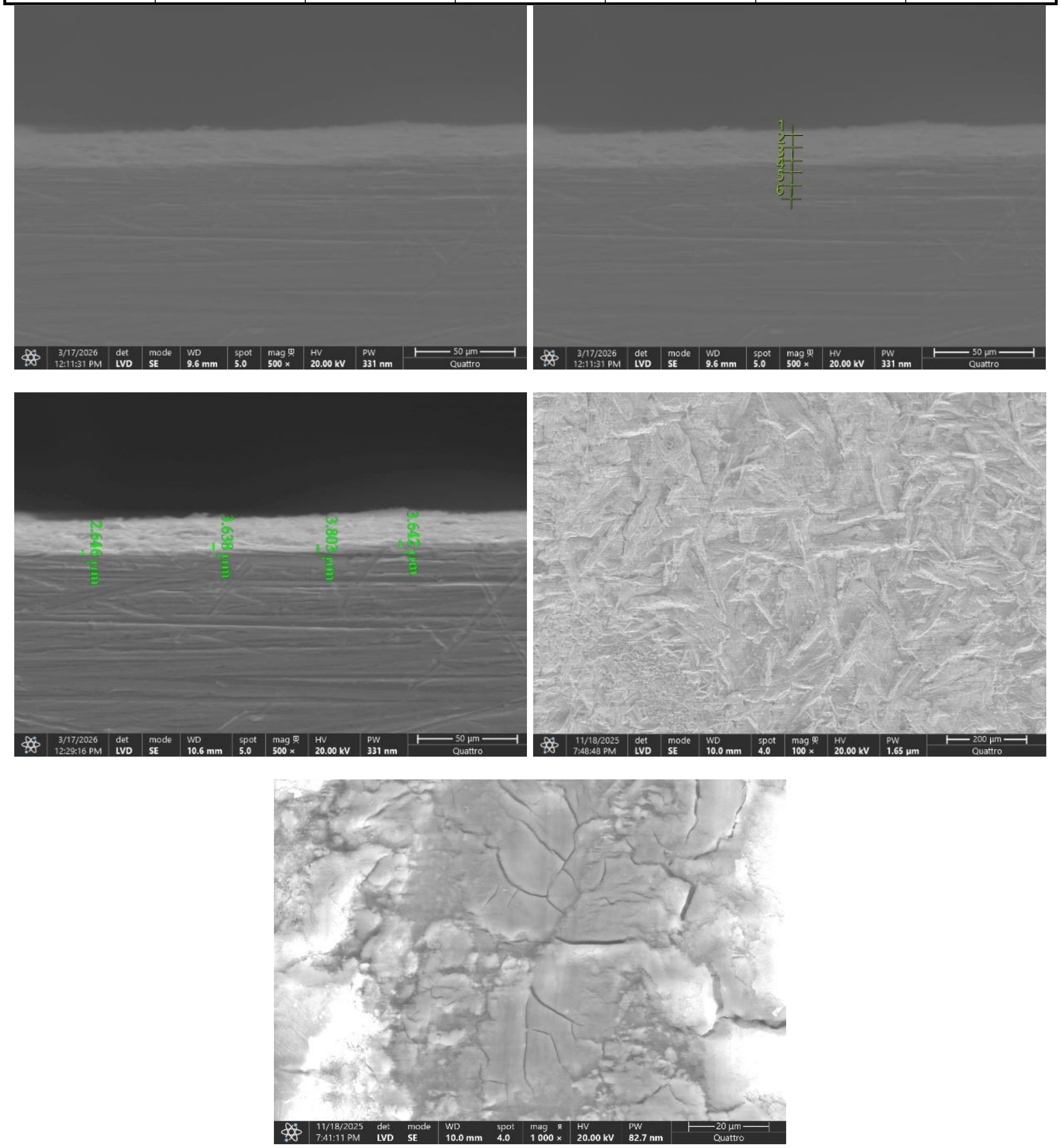

Figure S5. SEM images of sample II.2 (transversal view) + coating.

Table S5. Atomic percentage results for sample II.2.

	Atomic %					
**Element**	**Point 1**	**Point 2**	**Point 3**	**Point 4**	**Point 5**	**Point 6**
O	10.3	9.4	1.7	0.0	0.0	0.0
Mg	0.0	0.0	0.0	0.0	0.0	0.0
Al	11.8	18.8	5.2	8.2	9.4	8.8

	Atomic %					
**Element**	**Point 1**	**Point 2**	**Point 3**	**Point 4**	**Point 5**	**Point 6**
P	0.2	0.3	0.0	0.0	0.1	0.0
Ti	74.0	58.4	88.8	89.2	87.6	87.2
V	2.7	1.9	3.6	2.3	2.7	3.7
Fe	0.2	0.2	0.1	0.1	0.1	0.2
Zn	0.5	10.8	0.4	0.1	0.1	0.1
Zr	0.3	0.2	0.2	0.1	0.0	0.0

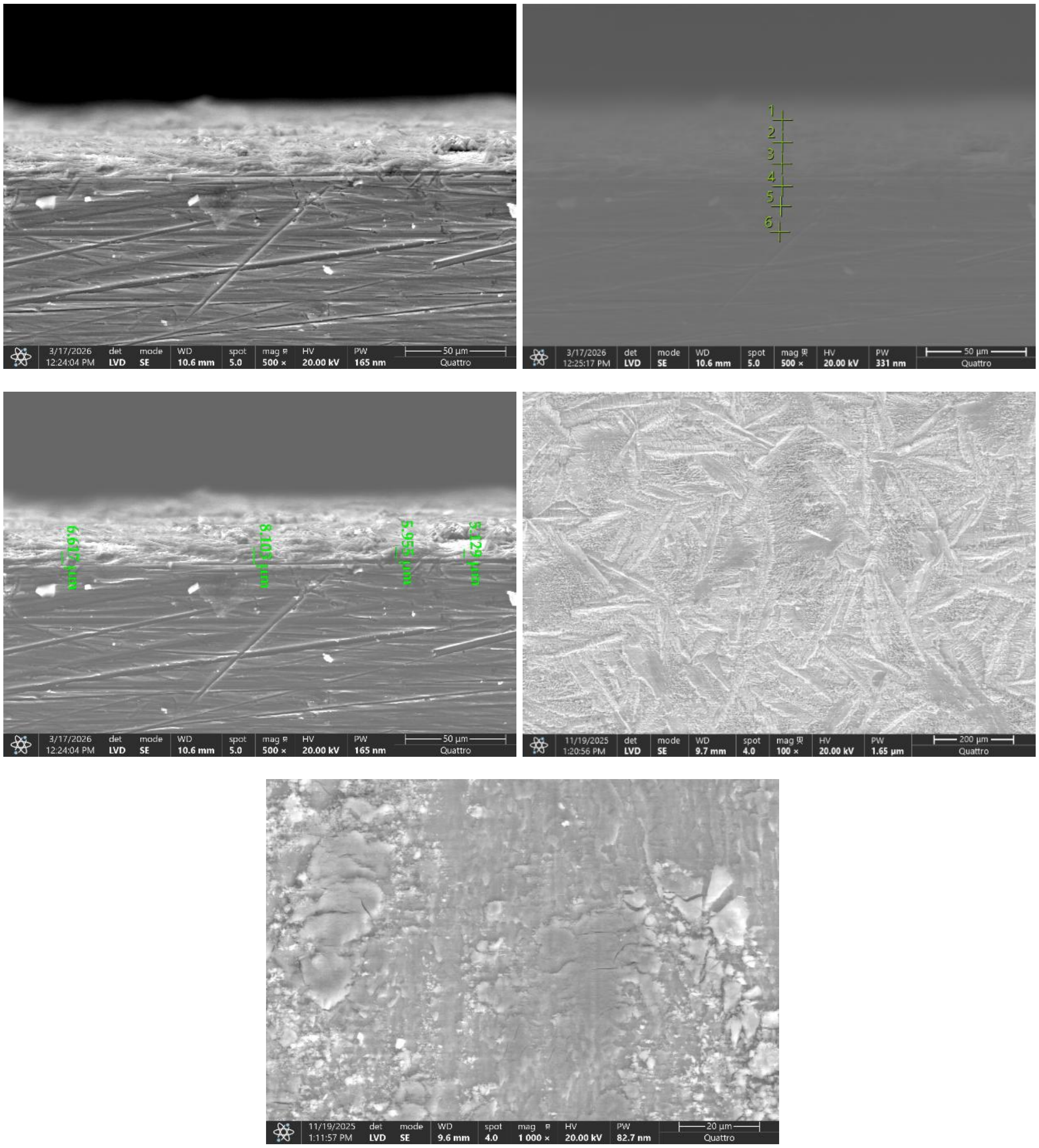

Figure S6. SEM images of sample II.3 (transversal view) + coating.

Table S6. Atomic percentage results for sample II.3.

	Atomic %					
**Element**	**Point 1**	**Point 2**	**Point 3**	**Point 4**	**Point 5**	**Point 6**
O	59.6	64.3	64.9	0.0	0.0	0.0
Mg	0.1	0.4	0.3	0.1	0.0	0.0
Al	18.4	6.2	8.0	14.1	13.8	13.7
P	6.3	8.7	4.6	0.5	0.3	0.0
Ti	7.5	8.0	10.4	81.3	82.3	81.9
V	0.3	0.3	0.5	2.9	2.5	2.7
Fe	0.2	0.1	0.1	0.1	0.2	0.1
Zn	7.5	12.0	10.0	0.7	0.7	0.6
Zr	0.1	0.0	1.2	0.3	0.2	1.0

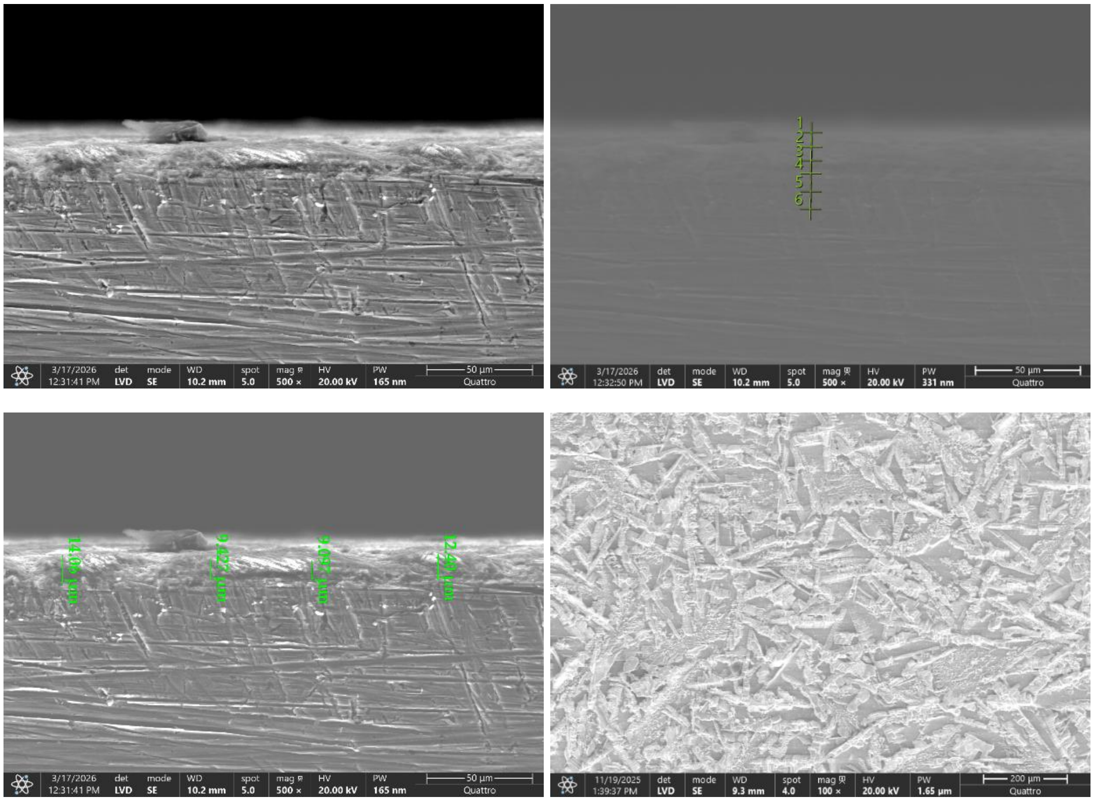

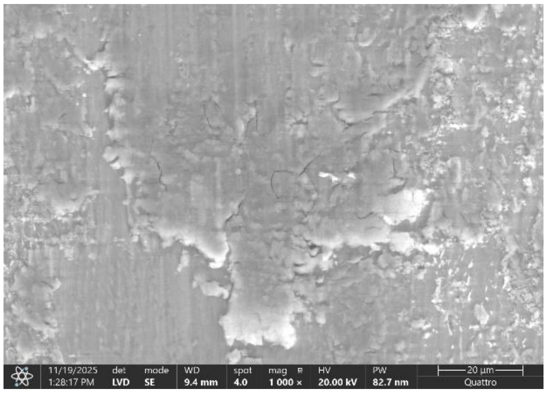

Figure S7. SEM images of sample III.1 (transversal view) + coating.

Table S7. Atomic percentage results for sample III.1.

	**Atomic %**					
**Element**	**Point 1**	**Point 2**	**Point 3**	**Point 4**	**Point 5**	**Point 6**
O	36.4	47.5	60.8	54.6	48.0	28.7
Al	34.9	22.5	8.2	9.4	8.7	9.8
P	0.1	1.7	6.8	0.0	0.3	0.0
Ca	0.2	0.5	0.2	0.3	0.0	0.6
Ti	21.2	12.9	10.5	27.3	37.4	56.9
V	0.9	0.5	0.4	1.0	1.3	3.1
Fe	0.2	0.2	0.2	0.2	0.1	0.2
Zn	4.1	10.9	11.5	4.7	3.3	0.3
Zr	2.0	3.3	1.4	2.5	0.9	0.4

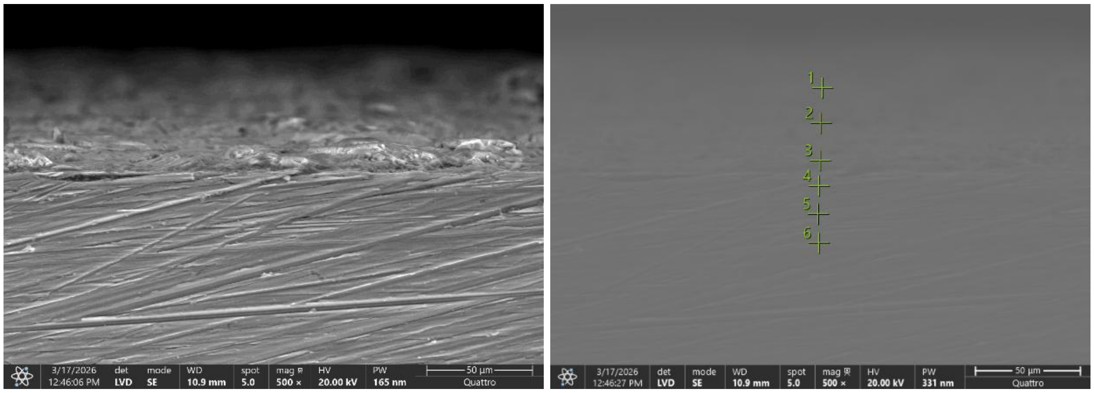

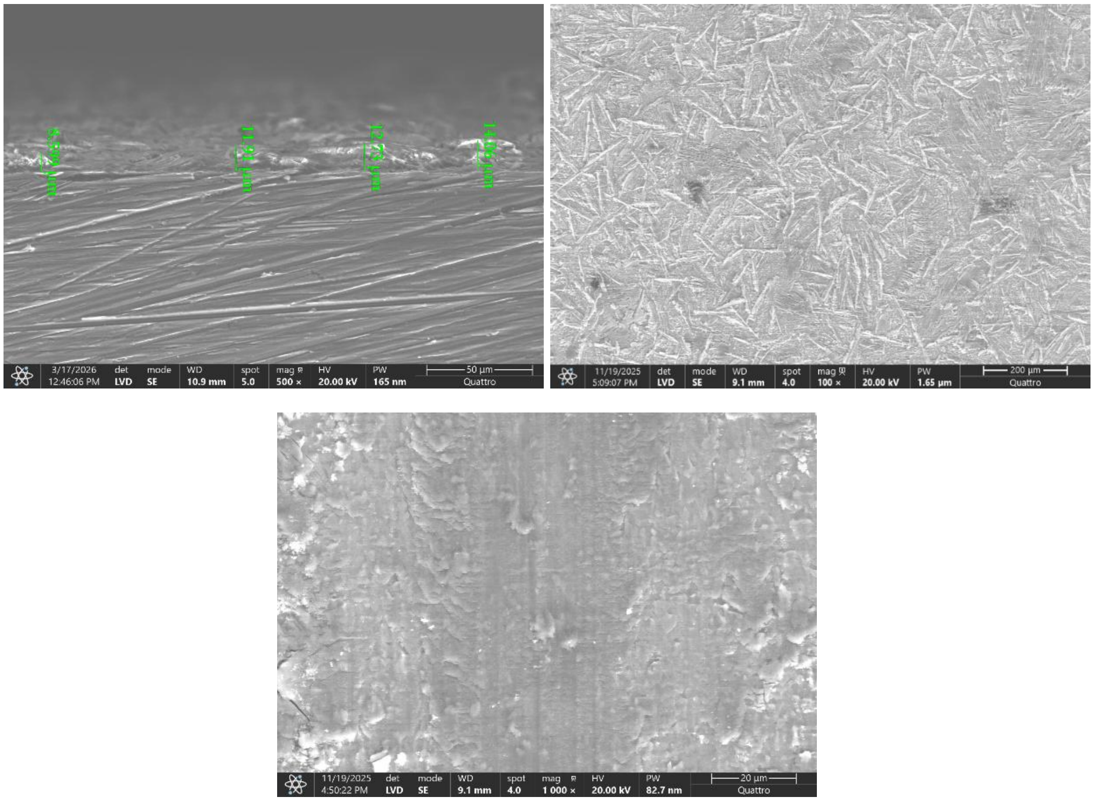

Figure S8. SEM images of sample III.2 (transversal view).

Table S8. Atomic percentage results for sample III.2.

	Atomic %					
**Element**	**Point 1**	**Point 2**	**Point 3**	**Point 4**	**Point 5**	**Point 6**
O	68.1	60.7	58.7	17.9	0.0	0.0
Al	14.4	13.9	11.3	18.9	21.1	20.6
P	6.3	8.0	2.0	0.5	0.4	0.2
Ca	0.1	0.1	0.2	0.0	0.0	0.0
Ti	4.2	6.6	10.0	59.4	75.1	76.0
V	0.2	0.3	0.4	1.8	2.2	2.3
Fe	0.3	0.6	0.3	0.2	0.1	0.1
Zn	6.4	8.5	1.8	0.9	0.8	0.6
Zr	0.0	1.3	15.3	0.4	0.3	0.2

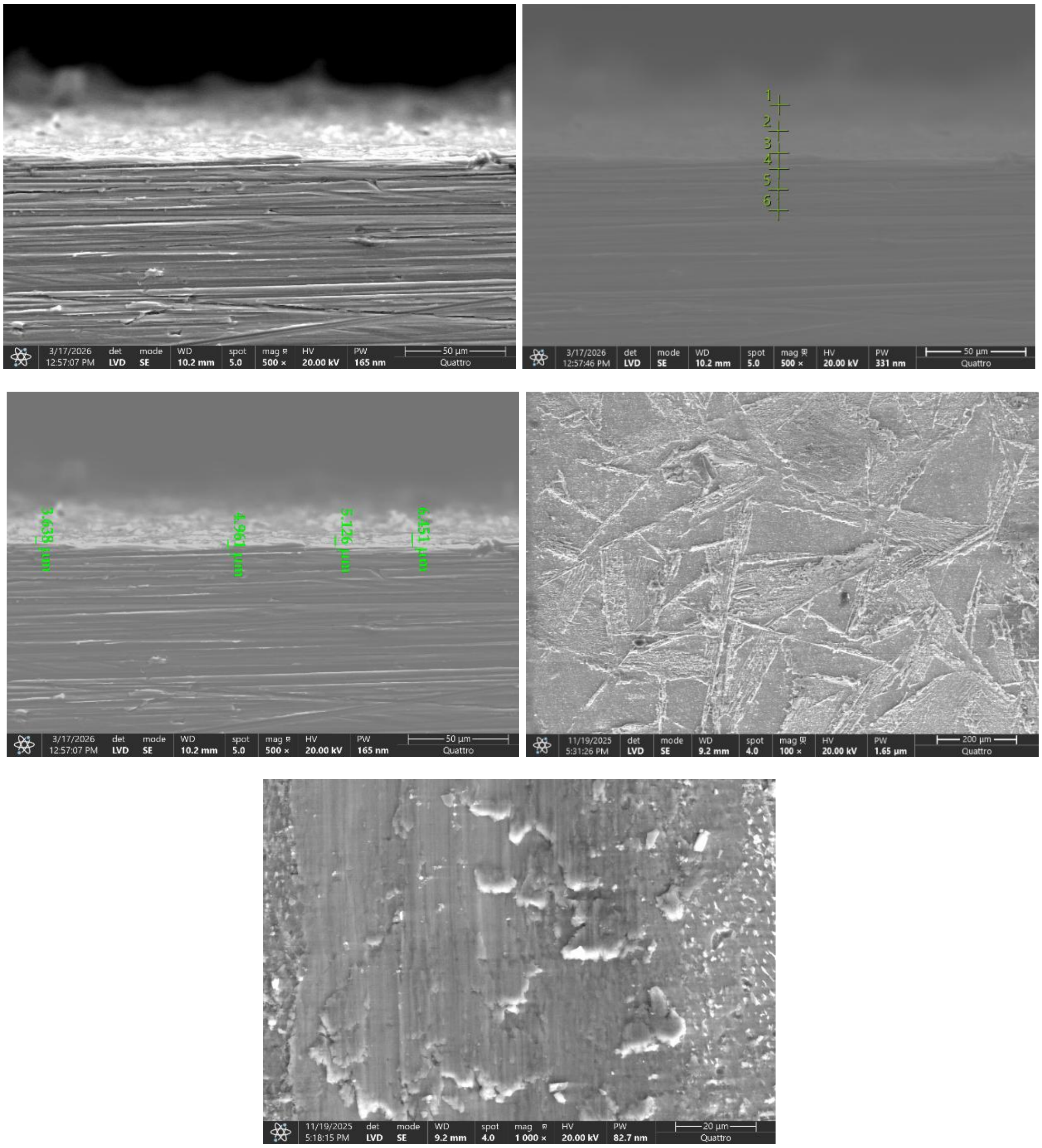

Figure S9. SEM images of sample III.3 (transversal view).

Table S9. Atomic percentage results for sample III.3.

	Atomic %					
**Element**	**Point 1**	**Point 2**	**Point 3**	**Point 4**	**Point 5**	**Point 6**
O	66.0	66.2	59.2	24.9	0.0	0.0
Al	2.9	2.9	3.4	6.9	9.1	9.5
P	2.3	2.5	2.0	0.1	0.0	0.0
Ca	0.0	0.0	0.3	0.3	0.0	0.0
Ti	25.5	24.7	28.7	65.6	87.7	87.8
V	0.9	0.8	1.1	1.7	2.8	2.4
Fe	0.1	0.1	0.2	0.2	0.1	0.1

	Atomic %					
**Element**	**Point 1**	**Point 2**	**Point 3**	**Point 4**	**Point 5**	**Point 6**
Zn	2.2	2.8	4.9	0.2	0.2	0.1
Zr	0.1	0.0	0.2	0.1	0.1	0.1

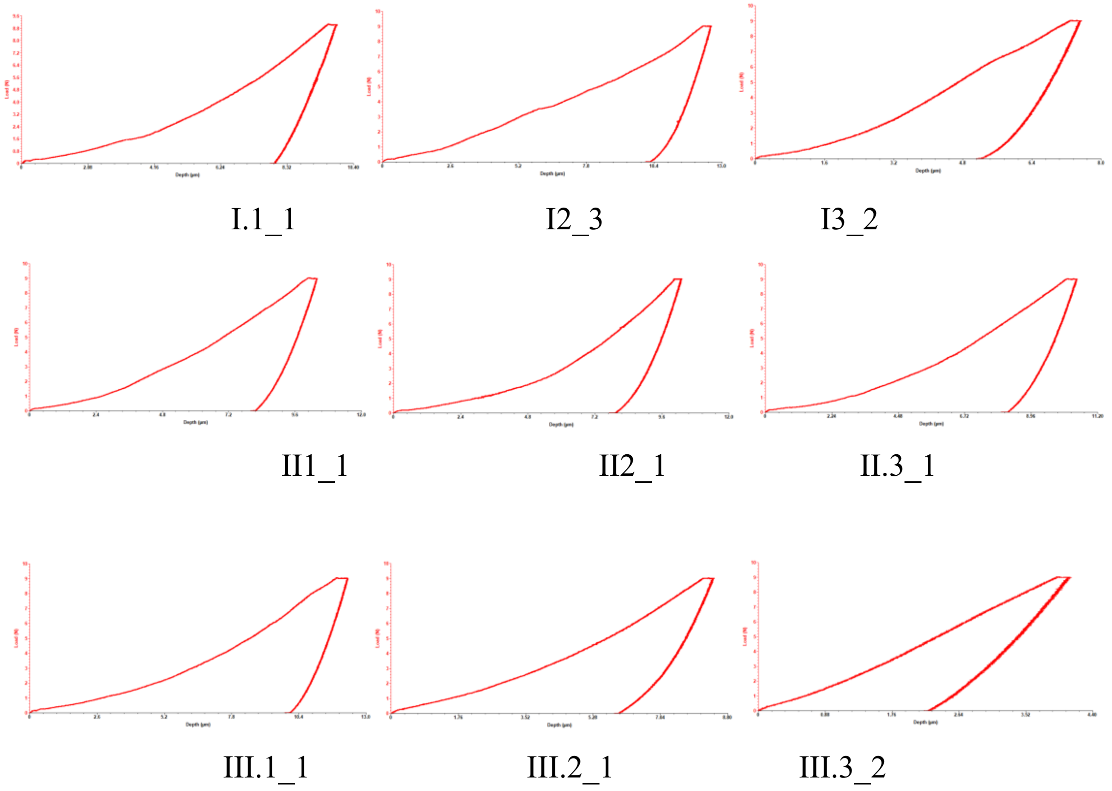

Figure S10. Indentation graphs for the phosphated samples.

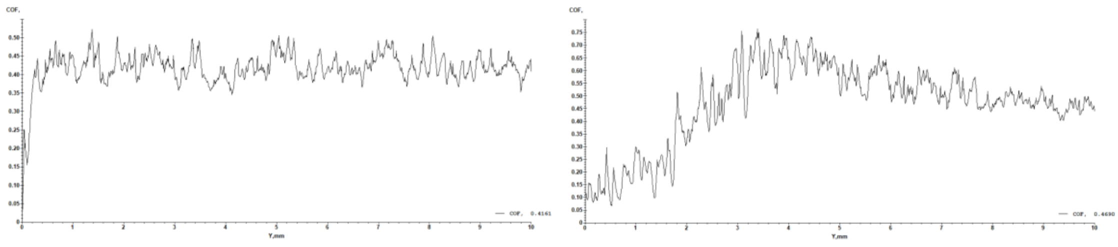

I.1_c(constant - 10N)   I.1_l(linear - 10N)

(a)

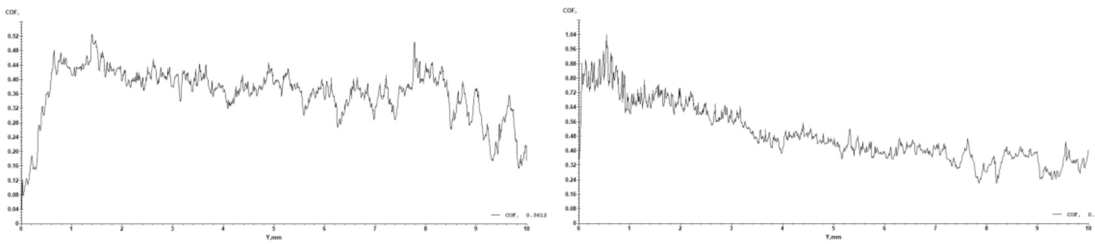

I.2_c I.2_l

(b)

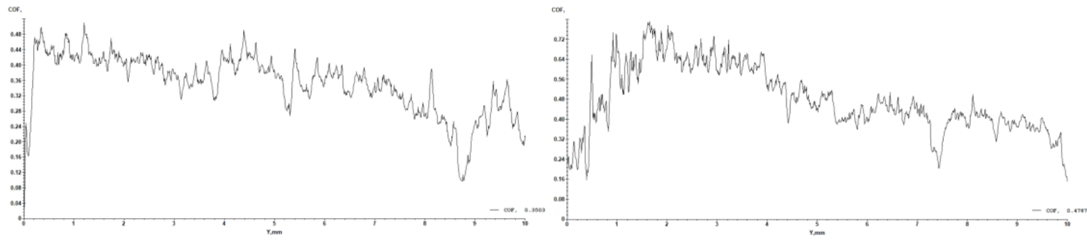

I.3_c I.3._l

(c)

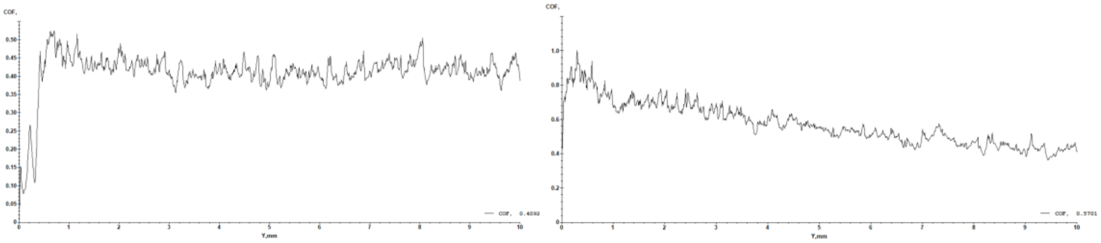

II.1_c II.1_l

(d)

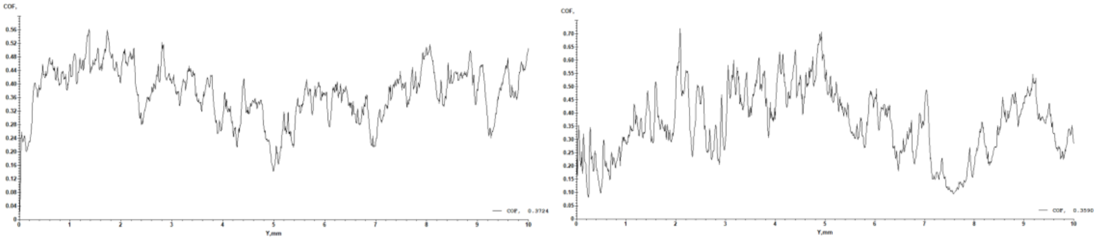

II.2_c II.2_l

(e)

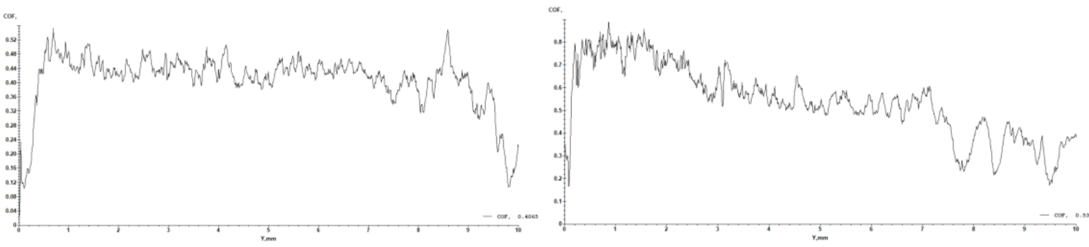

II.3_c II.3_l

(f)

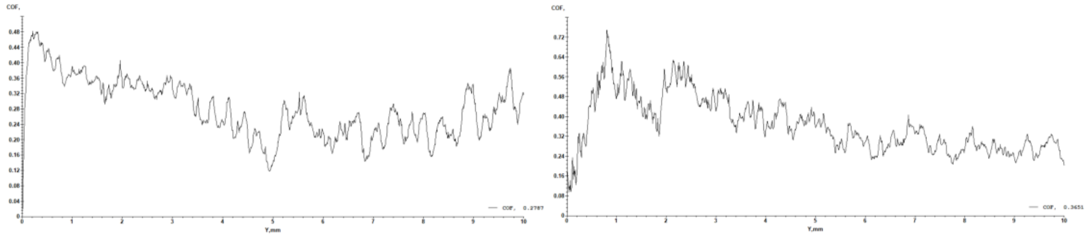

III.1_c III.1_l

(g)

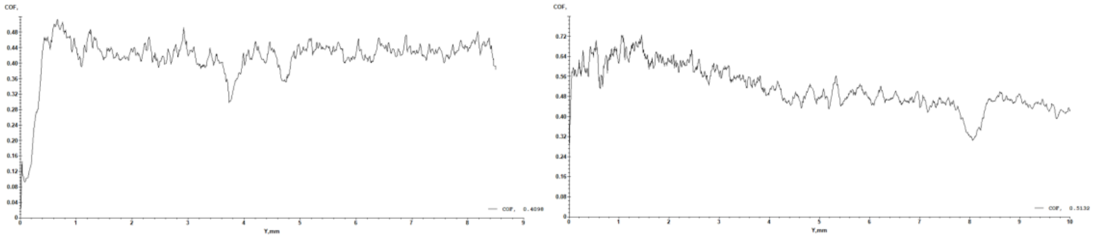

III.2_c III.2_l

(h)

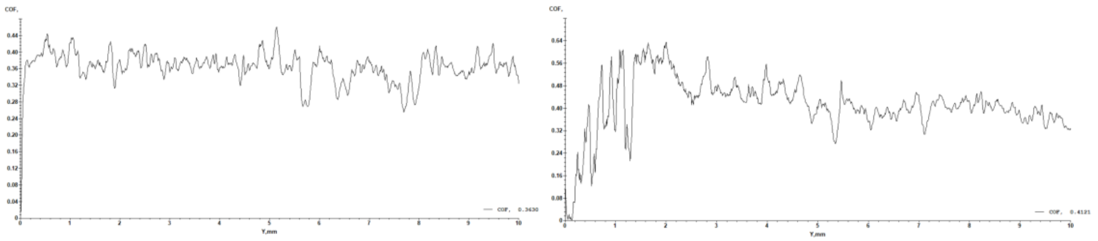

III.3.c III.3.l

(i)

Figure S11. Variations in the coefficient of friction (COF) over the 10 mm distance on which the scratch resistance tests were performed (with a constant or linear load of 10 N): (a) sample I.1, (b) I.2, (c) I.3, (d) II.1, (e) II.2, (f) II.3, (g) III.1, (h) III.2, and (i) III.3.

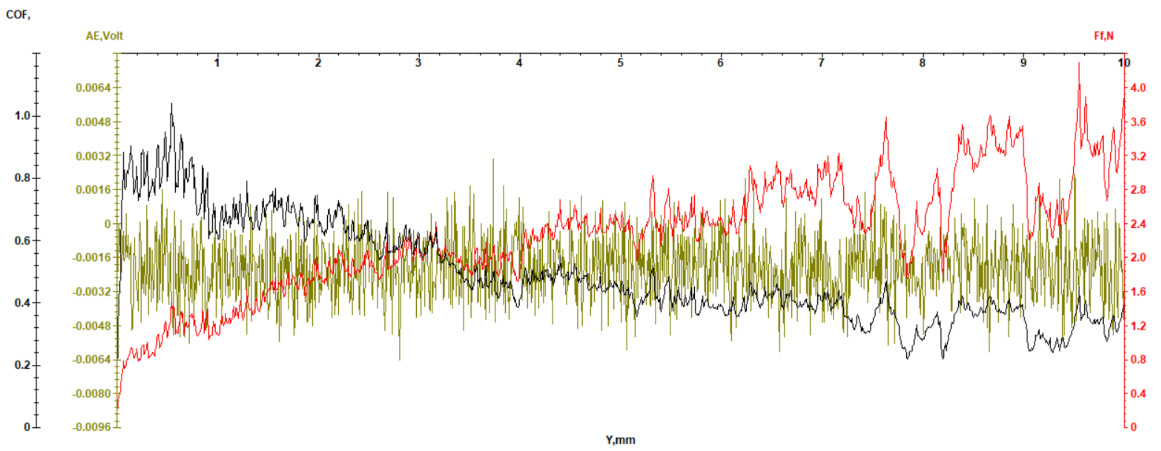

Figure S12. The I.2 sample's scratch plot.

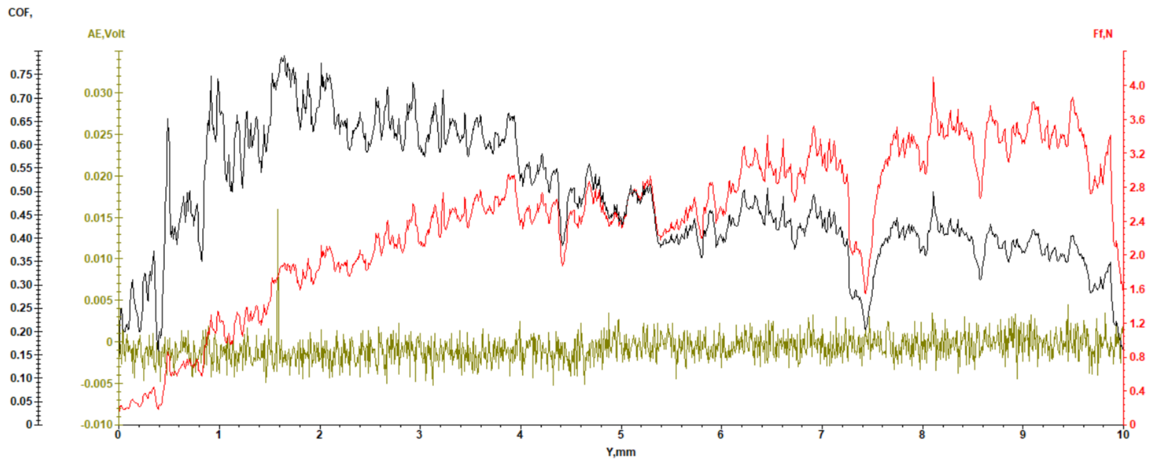

Figure S13. The I.3 sample's scratch plot.

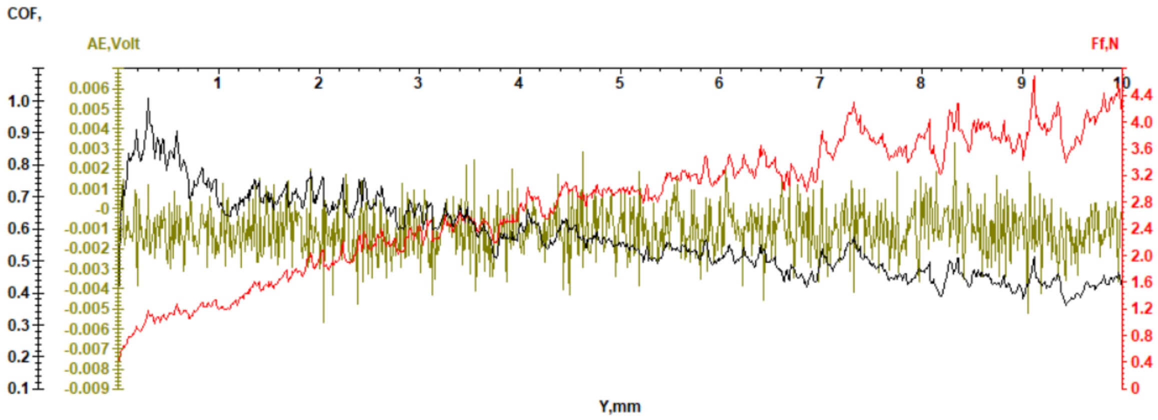

Figure S14. The II.1 sample's scratch plot.

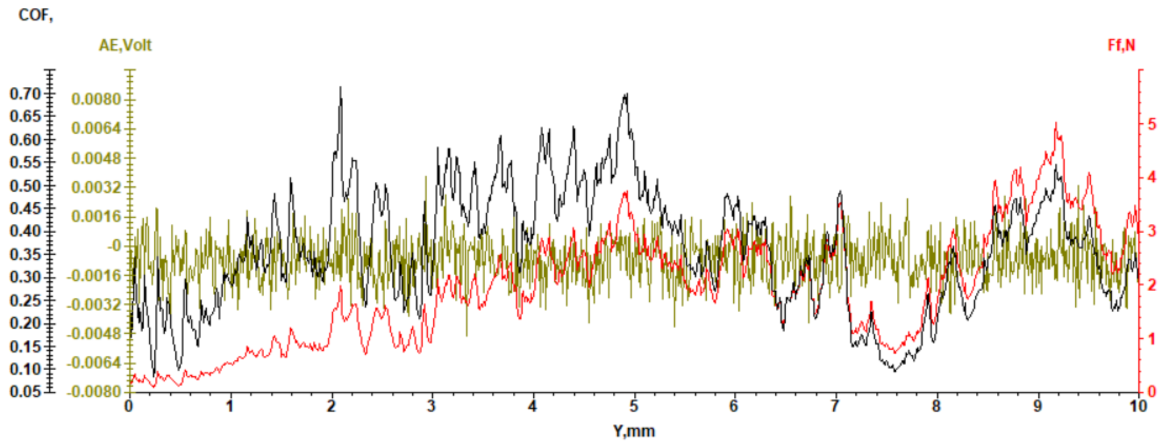

Figure S15. The II.2 sample's scratch plot.

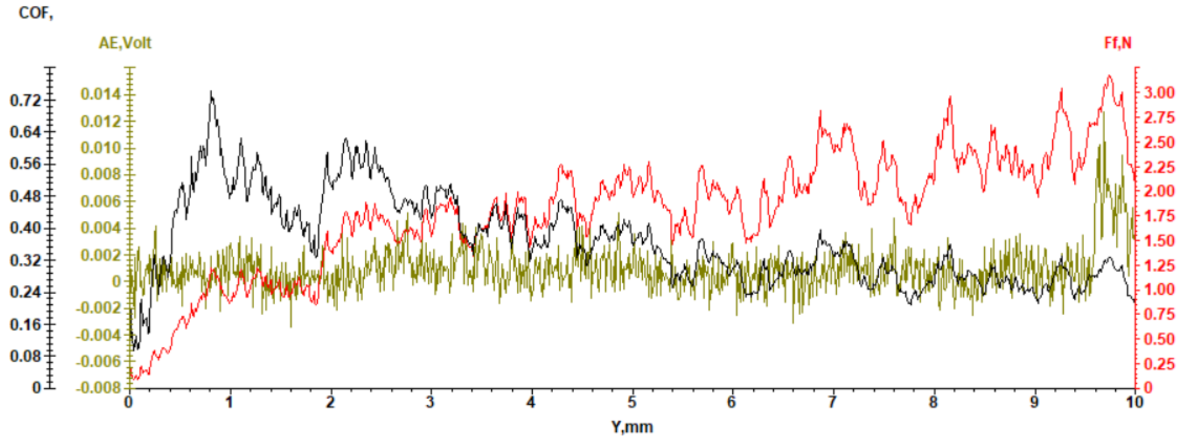

Figure S16. The III.1 sample's scratch plot.

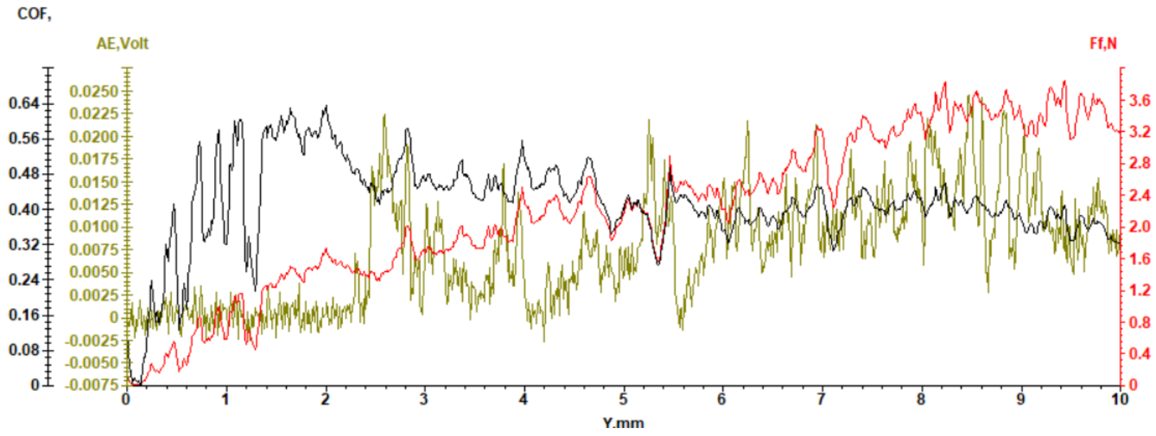

Figure S17. The III.3 sample's scratch plot.
